# Effects of Gamma Irradiation on Changes in Chemical Composition and Antioxidant Activity of *Euphorbia maculata* Callus

**DOI:** 10.3390/plants13162306

**Published:** 2024-08-19

**Authors:** Gyeong Han Jeong, Shubhpreet Kaur, Youngchul Yoo, Young Bae Ryu, Seo Jun Lee, Kwang-Woo Jung, Moon-Soo Chung, Hyoung-Woo Bai, Jin-Hong Kim, Sungbeom Lee, Tae Hoon Kim, Byung Yeoup Chung, Seung Sik Lee

**Affiliations:** 1 Advanced Radiation Technology Institute (ARTI), Korea Atomic Energy Research Institute (KAERI), Jeongeup 56212, Republic of Korea; jkh4598@kaeri.re.kr (G.H.J.); shubhpreetk49@gmail.com (S.K.); yooyc84@kakao.com (Y.Y.); kwjung@kaeri.re.kr (K.-W.J.); mschung@kaeri.re.kr (M.-S.C.); hbai@kaeri.re.kr (H.-W.B.); jhongkim@kaeri.re.kr (J.-H.K.); sungbeom@kaeri.re.kr (S.L.); bychung@kaeri.re.kr (B.Y.C.); 2 Functional Biomaterial Research Center, Korea Research Institute of Bioscience and Biotechnology (KRIBB), Jeongeup 56212, Republic of Korea; ybryu@kribb.re.kr (Y.B.R.); lji613@kribb.re.kr (S.J.L.); 3 Department of Radiation Science, University of Science and Technology (UST), Daejeon 34113, Republic of Korea; 4 Department of Food Science and Biotechnology, Daegu University, Gyeongsan 38453, Republic of Korea; skyey7@daegu.ac.kr

**Keywords:** *Euphorbia maculata*, gamma irradiation, plant-derived callus, radical scavenging, UPLC-QTOF/MS

## Abstract

In this study, we investigated the effects of gamma irradiation on the antioxidant activity and metabolite profiles of *Euphorbia maculata* calli (PC3012). Gamma irradiation at various doses (0, 0.05, 0.5, and 10 kGy) significantly enhanced the 1,1-diphenyl-2-picrylhydrazyl (DPPH) and 2,2′-azino-*bis*(3-ethylbenzothiazoline-6-sulphonic acid (ABTS^+^) radical scavenging activities of the callus extracts of PC3012 in a dose-dependent manner. High-performance liquid chromatography (HPLC) and ultra-performance liquid chromatography-quadrupole time-of-flight/mass spectrometry (UPLC-Q-TOF/MS) analyses revealed that irradiation increased the lysophospholipid content, although no new antioxidant compounds were formed. Furthermore, a PLS-DA analysis revealed evident metabolic differences between non-irradiated and irradiated samples, which were further verified by statistical validation. These findings suggest that gamma irradiation induces specific biochemical modifications that enhance the bioactive properties of PC3012 calli. This technology exhibits potential for utilization in the natural product and food sectors, particularly in the development of functional foods and nutraceuticals with improved health benefits.

## 1. Introduction

Studies were conducted approximately 30 years ago to enhance the production of secondary metabolites by inducing alterations in plant metabolic enzymes [1]. Several studies have demonstrated that irradiation under controlled conditions can result in beneficial hormonal responses in plants; therefore, exposure to low doses of radiation may have beneficial effects on plant growth [2]. Recent research is beginning to elucidate the molecular basis of these alterations, as well as their relationship with phenotypic changes. Heat shock proteins, the proteasome, activation of the kinase cascade, nitrogen metabolism, plant hormone balance, and antioxidant response mechanisms were all observed to undergo changes. It is possible that these alterations are the basis for hormonal responses in irradiated plants [3]. Gamma irradiation is a widely used technique for food preservation, sterilization, and the enhancement of plant-derived bioactive properties [4]. This process involves the exposure of substances to gamma rays, which can induce various biochemical changes, potentially leading to improved biological activities [5]. However, investigations into the formation of natural materials using such radiation are inadequate, and studies on the activity of secondary metabolites in many different plants are required. Recent research has shown that gamma irradiation can significantly alter plant metabolism, particularly in the regulation of secondary metabolites such as chlorophylls, carotenoids, and anthocyanins. For instance, in *Arabidopsis thaliana*, gamma irradiation has been linked to delayed senescence and enhanced photosynthetic efficiency by preserving the integrity of chloroplasts and thylakoid membranes [6,7]. These findings suggest that gamma irradiation not only serves as a mutagenic agent but also as a potential enhancer of plant resilience and bioactive compound production, thereby highlighting the need for further studies on its effects across various plant species.

*Euphorbia maculata*, a member of the family Euphorbiaceae, is extensively naturalized in Korea, Japan, and China [8]. This plant is characterized by soft, prostrate, and branched stems that spread along the ground, and the leaves have distinctive red spots on the central vein. Traditionally, *E. maculata* has been used to alleviate bleeding and various ailments, including epistaxis, wounds, diarrhea, dysentery, carbuncles, and warts [9,10]. Previous phytochemical investigations of *E. maculata* have led to the isolation of several novel compounds, such as triterpenoids, flavonoids, tannins, and phenolic acids [11,12]. Pharmacological studies have confirmed its wide variety of biological activities, including antibacterial, antifungal, antioxidant, anti-inflammatory, anti-osteoporotic, anticancer, antiplatelet, anti-tyrosinase, and anti-obesity effects [13]. However, there have been no studies on the functionality and primary ingredients of calli.

Callus culture, the in vitro cultivation of plant cells, offers a controlled environment for studying and enhancing the production of valuable phytochemicals [14]. This technique is crucial for investigating the biosynthetic pathways and stress responses of plants, leading to the development of novel compounds with enhanced bioactivity. Callus cultures are extensively used in a variety of research disciplines, including plant physiology, biochemistry, and pharmacology, and are also employed in the production of medicinal compounds, genetic modifications, and the conservation of rare plant species [15]. In the present study, we investigated the effects of gamma irradiation on the antioxidant activity and metabolite profiles of *E. maculata* callus. By employing assays such as 1,1-diphenyl-2-picrylhydrazyl (DPPH) and 2,2′-azino-*bis*(3-ethylbenzothiazoline-6-sulphonic acid (ABTS^+^) radical scavenging activities, along with an advanced ultra-performance liquid chromatography-quadrupole time-of-flight/mass spectrometry (UPLC-Q-TOF/MS) analysis, we aimed to elucidate the specific biochemical changes induced by gamma irradiation. This study aimed to contribute to the growing body of knowledge on the use of irradiation to enhance the functional properties of natural products, with potential applications in the development of functional foods and nutraceuticals.

## 2. Results

### 2.1. Radical Scavenging Ability of Plant-Derived Callus by Gamma Irradiation

**Figure 1:**
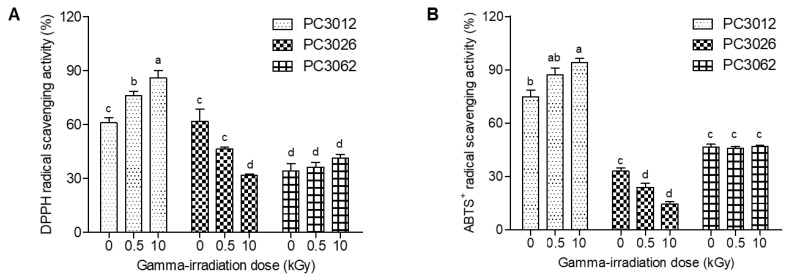
DPPH and ABTS^+^ radical scavenging activities of three plant-derived calli after gamma irradiation. (**A**) DPPH radical scavenging assay; (**B**) ABTS^+^ radical scavenging assay. Radical scavenging ability was measured at 100 μg/mL. The results are expressed as mean ± SD (*n* = 3). Statistical analyses were performed using a two-way ANOVA, followed by Tukey’s post hoc test. Different letters represent statistically significant differences at *p* < 0.05. PC0312: *E. maculata* callus; PC3026: *P. granatum* callus; PC3062: *P. fragarioides* callus.

Plant-derived callus cultures are valuable because of their ability to produce high concentrations of bioactive compounds [16]. In this study, we evaluated the radical scavenging activities of three plant-derived calli (*E. maculata*, *Punica granatum*, and *Potentilla fragarioides*) after gamma irradiation. Figure 1 shows that gamma irradiation significantly affects the antioxidant activities of the calli, as evidenced by changes in DPPH and ABTS^+^ radical scavenging activities at a concentration of 100 μg/mL. The DPPH radical scavenging activity of *E. maculata* calli (PC0312) increased from 61.0% to 85.9%, whereas that of *P. fragarioides* calli (PC3062) slightly improved from 34.1% to 41.4%. Conversely, the DPPH radical scavenging activity of *P. granatum* calli (PC3026) decreased from 61.8% to 31.8% (Figure 1A). Similarly, the ABTS^+^ radical scavenging activity of the PC3012 extract increased from 74.8% to 94.0% (Figure 1B). Among the three callus extracts, *E. maculata* callus (PC0312) exhibited the highest increase in both DPPH and ABTS^+^ scavenging activities, indicating greater sensitivity to gamma irradiation.

### 2.2. Radical Scavenging Ability of E. maculata Callus after Different Doses of Gamma Irradiation

**Figure 2:**
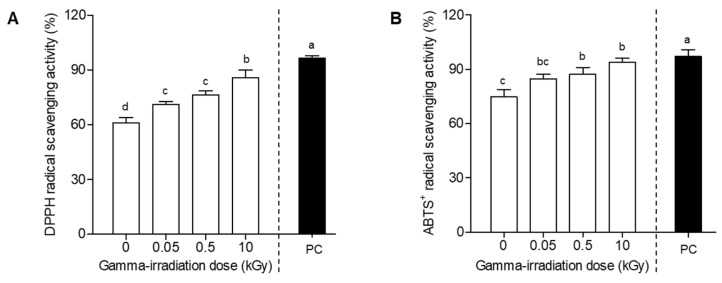
DPPH and ABTS^+^ radical scavenging activities of *E. maculata* callus extracts at gamma radiation doses of 0, 0.05, 0.5, and 10 kGy. (**A**) DPPH radical scavenging assay; (**B**) ABTS^+^ radical scavenging assay. Radical scavenging ability was measured at 100 μg/mL. The results are expressed as mean ± SD (*n* = 3). Statistical analyses were performed using a two-way ANOVA, followed by Tukey’s post hoc test. Different letters represent statistically significant differences at *p* < 0.05. PC: (+)-Catechin used as a positive control.

*E. maculata* extracts have shown a diverse range of functional properties, including antioxidant, anti-inflammatory, and antimicrobial activities [17]. In our study, *E. maculata* extracts exhibited the greatest increase in antioxidant activity (Figure 1). However, research related to *E. maculata* calli is scarce. In this experiment, we evaluated the antioxidant activities of *E. maculata* calli (PC3012) exposed to various gamma irradiation doses. Figure 2 depicts the antioxidant activity of *E. maculata* calli (PC3012), at different gamma irradiation doses (0, 0.05, 0.5, and 10 kGy). The DPPH radical scavenging activity increased from an initial value of 61.0% to 70.9%, 76.2%, and 85.9% at 0.05, 0.5, and 10 kGy of gamma irradiation, respectively, showing a clear dose-dependent activity enhancement (Figure 2A). Similarly, the ABTS^+^ radical scavenging activity increased from 74.8% at 0 kGy to 94.0% at 10 kGy, indicating a significant improvement in antioxidant capacity with increasing gamma irradiation doses (Figure 2B). To further understand the specific changes in antioxidant compounds, we hypothesized that gamma irradiation modifies key phytochemicals within the callus. Consequently, we performed HPLC and UPLC-Q-TOF/MS analyses to identify and characterize the specific substances that have changed after irradiation.

### 2.3. High-Performance Liquid Chromatography (HPLC) Analysis of E. maculata Callus by Gamma Irradiation

**Figure 3:**
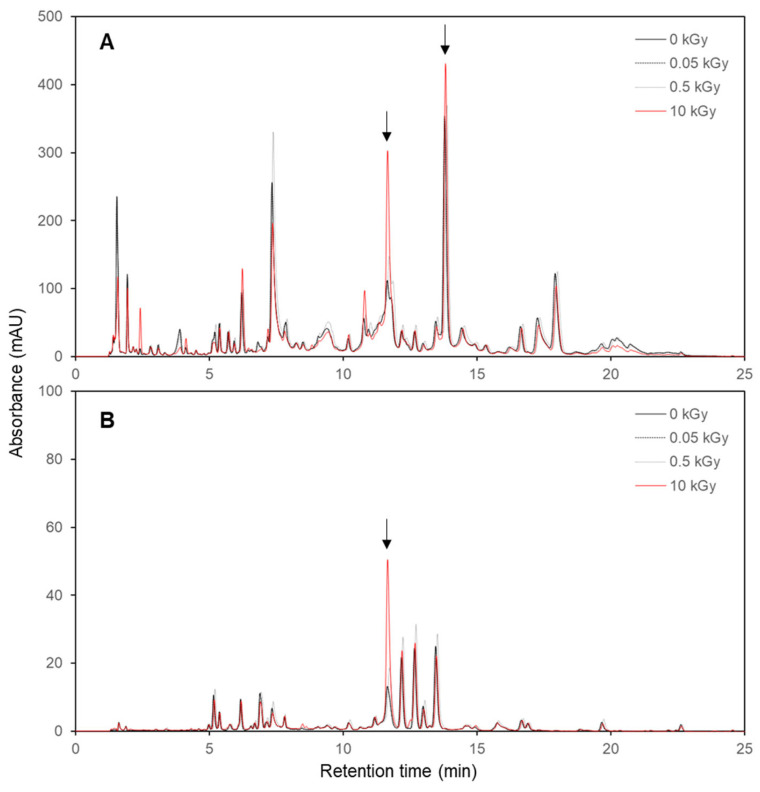
HPLC chromatograms of non-irradiated and irradiated *E. maculata* (PC3012) callus extracts. (**A**) 254 nm; (**B**) 360 nm. See the Materials and Methods section for experimental conditions. Arrow shows the increased peak by gamma irradiation in plant callus.

A high-performance liquid chromatography (HPLC) analysis revealed significant differences in the chemical profiles of the non-irradiated and irradiated *E. maculata* callus extracts. The 10 kGy irradiated samples exhibited higher peak intensities and additional peaks at 254 nm (Figure 3A) and 360 nm (Figure 3B) than the non-irradiated and lower gamma-dose samples. No significant difference was observed between the calli extracts exposed to low irradiation doses (0.05 and 0.5 kGy) and the non-irradiated sample. The 10 kGy samples exhibited more pronounced peaks at 11.6 and 13.8 min retention times, indicating that higher irradiation doses contribute to an increase in specific compounds. These results suggest that the gamma irradiation induced changes in the metabolite profiles of the callus. The increased peak intensities and appearance of new peaks in the irradiated samples imply the enhanced production of certain phytochemicals and the formation of novel compounds. These changes enhanced the bioactive properties of the extracts.

### 2.4. Identification of Compounds Produced in E. maculata Callus by Gamma Irradiation

**Figure 4:**
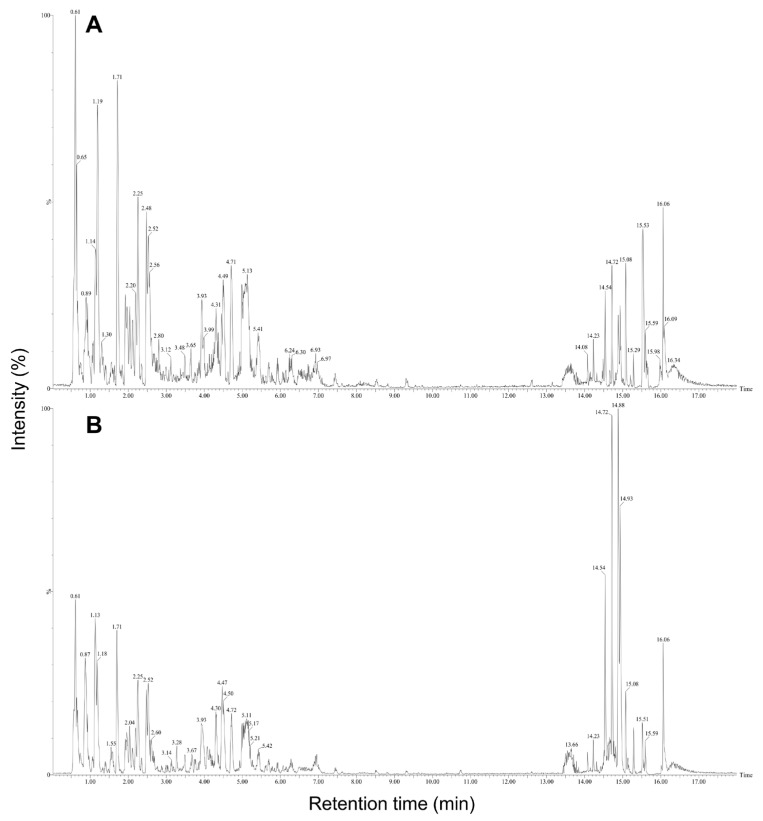
TIC chromatograms of non-irradiated (**A**) and irradiated (**B**) *E. maculata* calli (PC3012). Refer to the Materials and Methods section for experimental conditions.

**Figure 5:**
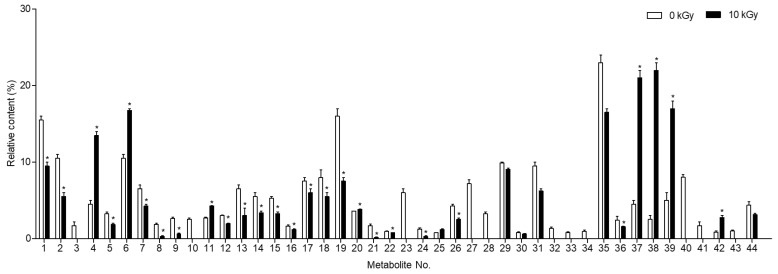
Changes in metabolite content (%) of non-irradiated and irradiated *Euphorbia maculata* calli (PC3012). The results are expressed as the mean ± standard deviation (*n* = 3). * *p* < 0.05 vs. the non-irradiated groups. Refer to Metabolite number in Table 1.

The predominant compounds in plant-derived calli of *E. maculata* (Figure 4) were identified using UPLC-Q-TOF/MS. The major metabolites were identified by comparing their retention times and MS data with those of authentic reference materials and previously reported data (Table 1). Moreover, the MS databases HMDB, METLIN, and ChemSpider were used to confirm the results (Figure S1). A total of 44 metabolites were identified in *E. maculata* callus, including 9 amino acid derivatives (_L_-arginine, *N*-(1-deoxy-1-fructosyl)isoleucine, γ-glutamyl lysine, *N*-(1-deoxy-1-fructosyl)phenylalanine, _L_-phenylalanine, γ-glutamylglutamic acid, *N*-(1-deoxy-1-fructosyl)tryptophan, _L_-trytophan, and phenylalanylglycine), 2 sesquiterpenoids (pteroside B and acetylpterosin C), 16 flavonoids (epicatechin 3-glucuronide, 2′,7-dihydorxy-4′,5′-dimethoxyisoflavone, epicatechin 4′-glucuronide, kaempferol 3-rhamnosyl-6″-(4″-acetylrhamnosyl)glucoside, leucocyanidin, kaempferol 3-rhamnosyl-(6″-acetyl)galactosyl-7-glucoside, 7-glucosyl-4″glucuronoyl epigallocatechin gallate, quercetin 3,7-diglucosyl-4″-galactoside, isoquercitrin, quercetin 3-coumaroyl-triglucoside, quercetin 3-(2‴,6‴-digalloyl)galactoside, quercetin 3-(2-galloyl)glucoside, isorhamnetin 3-rutinoyl-4′-rhamnoside, kaempferol 3-(2″-rhamnosyl-6″-acetyl)galactosyl-7-rhamnoside, kaempferol 3-feruloyl-triglucoside, and isooreientin), 7 phenolic acids (sinapic acid, gallic acid 3-*O*-gallate, quinic acid, chlorogenic acid, methyl gallate, caffeic acid ethyl ester, and 1,2,3,4,6-pentagallolyglucose), 7 lipids (dehydrophytosphingosine, lysoPC(18:3), lysoPC(18:2), lysoPC(16:0), lysoPC(18:1), phytosphingsine 1-phosphate, and PC(18:3/18:3)), and 3 chlorophylls (methyl phaephoribide B, pheophorbide A, and pheophorbide B). However, the gamma-irradiated *E. maculata* callus contained only 35 metabolites, excluding γ-glutamylglutamic acid, leucocyanidin, isoquercitrin, quercetin 3-coumaroyl-triglucoside, kaempferol 3-(2″-rhamnosyl-6″-acetyl)galactosyl-7-rhamnoside, kaempferol 3-feruloyl-triglucoside, isooreientin, phytosphingsine 1-phosphate, and methyl phaephoribide B (Table 1). After gamma irradiation, the content of the majority of phenolic derivatives (including flavonoids and phenolic compounds) decreased, but the content of γ-glutamyl lysine, *N*-(1-deoxy-1-fructosyl)phenylalanine, *N*-(1-deoxy-1-fructosyl)tryptophan, lysoPC(18:2), lysoPC(16:0), and lysoPC(18:1) significantly increased by 9.0%, 5.4%, 1.9%, 16.6%, 20.4%, and 12.9%, respectively (Figure 5).

### 2.5. Statistical Analysis of Changes in E. maculata Calli after Gamma Irradiation

**Figure 6:**
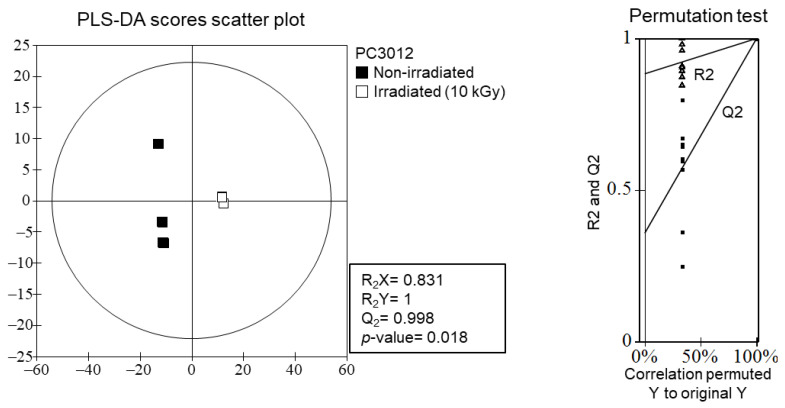
PLS-DA scores scatter plot and permutation test analysis of metabolite profiles of non-irradiated and irradiated *E. maculata* calli (PC3012). △: R2 values, ■: Q2 values.

The PLS-DA score scatter plot and permutation test analyses (Figure 6) confirmed the significant effects of gamma irradiation on the metabolite profiles of *E. maculata* calli. The scatter plot revealed a distinct separation between non-irradiated and irradiated (10 kGy) samples, indicating substantial differences in their metabolite compositions. The permutation test supported the robustness and statistical significance of the PLS-DA model, with high R_2_X (0.831) and Q2 (0.998) values and a *p*-value of 0.018 (Table S1), which confirm that the observed metabolic changes were not due to random variation but were induced by gamma irradiation.

## 3. Discussion

The primary objective of this experiment was to investigate the potential of gamma irradiation to enhance the antioxidant properties and modify the metabolite profiles of *Euphorbia maculata* callus. This study builds on previous research suggesting that gamma irradiation can induce beneficial biochemical changes in plant tissues, making it a valuable tool for improving the bioactive characteristics of plant-derived materials. The three callus extracts derived from *E. maculata*, *Punica granatum*, and *Potentilla fragarioides* exhibited different responses to gamma irradiation. While the antioxidant activity of the *E. maculata* callus (PC0312) significantly increased, as shown by the DPPH and ABTS^+^ assays, the *P. granatum* callus (PC3026) displayed a decrease in antioxidant activity, and the *P. fragarioides* callus (PC3062) showed only a slight improvement. These differential responses suggest that the impact of gamma irradiation on antioxidant activity is highly dependent on the specific metabolic pathways and the inherent chemical composition of each plant species. In the case of *E. maculata*, the increase in antioxidant activity may be attributed to the upregulation of specific enzymes or the enhanced synthesis of antioxidant compounds such as flavonoids and phenolic acids, which are known to be sensitive to radiation-induced stress [18]. The callus of *E. maculata* may have a robust defense mechanism that is activated under gamma irradiation, leading to an increase in the production of antioxidant metabolites. On the other hand, the decrease in antioxidant activity observed in the *P. granatum* callus could be due to the degradation of sensitive antioxidant compounds such as certain polyphenols and flavonoids, which may break down under gamma irradiation into less active or even inactive forms. This degradation might outweigh any potential increase in antioxidant activity from other newly synthesized compounds [19]. Additionally, *P. granatum* may have less efficient stress response pathways compared to *E. maculata*, leading to a net decrease in antioxidant potential following irradiation. For *P. fragarioides*, the slight improvement in antioxidant activity suggests a balance between the degradation of some antioxidants and the synthesis of others. This balance may result in a marginal increase that is not as pronounced as that observed in *E. maculata*, but still represents an enhancement over the non-irradiated state. It is possible that while certain metabolites are degraded, others are either synthesized or their activity is enhanced, leading to a slight net gain in antioxidant capacity. These findings underscore the complexity of plant responses to gamma irradiation, highlighting that the effects are not uniform across different species or even within the same metabolic pathways. The variability in response could be influenced by several factors, including the specific types of metabolites present, the structure of the callus tissues, and the efficiency of the plant’s inherent stress response mechanisms. Furthermore, these results suggest that gamma irradiation may be selectively applied depending on the desired outcome, whether it is to enhance or modify the antioxidant properties of specific plant extracts. Overall, this study reveals that while gamma irradiation can be a powerful tool for enhancing the bioactive properties of certain plant calli, its effects are not universally positive and must be carefully evaluated for each specific application. Further research is warranted to explore the molecular mechanisms underlying these differential responses and to optimize irradiation conditions to achieve the desired bioactive enhancements in various plant-derived materials

The dose-dependent increase in both DPPH and ABTS^+^ radical scavenging activities of PC3012 calli, as shown in Figure 2, further emphasizes the effectiveness of gamma irradiation in enhancing antioxidant characteristics. This suggests that gamma irradiation stimulates the synthesis or activation of antioxidant compounds in calli, with higher doses resulting in greater antioxidant activity. These findings highlight the viability of gamma irradiation as an approach to enhancing the bioactive properties of plant-derived materials, making them beneficial for pharmaceutical and nutraceutical applications [20]. Previous studies have reported that gamma irradiation affects the growth of plant-derived callus [21]. However, in this study, extraction was performed immediately after gamma irradiation without any time delay. In addition, since gamma irradiation was performed at a dose rate of 10 kGy/h, the growth time was only about 1 h at most. Therefore, it is judged that gamma irradiation did not affect the growth inhibition of callus tissue. While our current study focused on a maximum irradiation intensity of 10 kGy, we acknowledge that there is potential for further enhancement of radical scavenging activities at higher doses. This consideration has been incorporated into our discussion, and we intend to conduct future research to explore the effects of higher irradiation intensities in order to determine the optimal conditions for maximizing antioxidant activity. By exploring higher irradiation doses, we aim to gain deeper insights into the relationship between irradiation and antioxidant capacity, which could lead to the development of more effective strategies for enhancing the bioactive properties of plant-derived materials.

The changes in metabolite content before and after gamma irradiation, as illustrated in Figure 5, indicate that gamma irradiation induces significant biochemical modifications in *E. maculata* calli. The enhanced antioxidant activity observed in irradiated calli may be attributable to these biochemical changes. Previous studies have reported that flavonoid derivatives are easily degraded by gamma irradiation, leading to the formation of compounds with strong antioxidant activity [22]. However, no new compounds were detected in the present study. Notably, we observed a substantial increase in lysophospholipid content after gamma irradiation. This finding aligns with previous research indicating that plants produce large amounts of lysophospholipids as a defense mechanism when subjected to excessive stress [23]. This suggests that gamma irradiation induces a stress response in plant calli, which results in an increase in the levels of lipid-based metabolites. To gain a deeper understanding of these changes, we performed UPLC-Q-TOF/MS analysis on the metabolite profile of *E. maculata* calli after gamma irradiation. The comprehensive analysis revealed significant changes in metabolite content that could be harnessed for practical applications. The increase in lysophospholipid content and the overall enhancement of antioxidant activity suggest that gamma irradiation can be used to improve the bioactive compounds of plant-derived calli. Previous studies have identified lysophospholipids in a wide range of tissues and cell types, where they play critical roles in various physiological functions, including vascular development, reproduction, myelination, neurological disorders, and cancer [24]. Consequently, gamma irradiation has the potential to enhance the antioxidant properties of materials, which can be beneficial for applications in the natural product and food industries [25].

The significant distinction in metabolite profiles between non-irradiated and irradiated *E. maculata* calli, as shown in Figure 6, suggests that gamma irradiation induces specific biochemical changes. While previous studies suggested that gamma irradiation can degrade flavonoid derivatives, resulting in the formation of lead compounds [22,26], our study did not detect new compounds. Instead, we observed a substantial increase in lysophospholipid content, consistent with the stress responses of plants. LC-Q-TOF/MS analysis confirmed these changes, highlighting the potential of gamma irradiation to enhance the bioactive properties of plant-derived materials. By optimizing the gamma irradiation conditions, it may be possible to create plant extracts with superior health benefits, making this a valuable technique for developing functional foods and nutraceuticals. Our study contributes to the growing body of evidence supporting the use of gamma irradiation to enhance the functional properties of natural products, thereby opening new opportunities for their application in various fields.

## 4. Materials and Methods

### 4.1. Chemicals

1,1-Diphenyl-2-picrylhydrazyl (DPPH) radical, 2,2′-azino-*bis*(3-ethylbenzothiazoline-6-sulphonic acid (ABTS^+^), methanol (MeOH), acetonitrile (MeCN), formic acid (HCOOH), and (+)-catechin were purchased from Sigma-Aldrich (St. Louis, MO, USA). All other chemicals used in this study were of analytical grade.

### 4.2. Gamma Irradiation of Plant-Derived Calli

Three plant-derived calli, namely *Euphorbia maculata* (PC3012), *Punica granatum* (PC3026), and *Potentilla fragarioides* (PC3062), were obtained from the Korean Collection for Type Cultures (KCTC) at the Korea Research Institute of Bioscience and Biotechnology (KRIBB) for use in the experiments (Figure S2). Gamma irradiation was conducted at room temperature following a previously established method using a cobalt-60 experimental irradiator (point source: AECL, IR-79, MDS Nordion International Co., Ltd., Ottawa, ON, Canada) at the Advanced Radiation Technology Institute, Korea Atomic Energy Research Institute, Jeongup, Republic of Korea [27]. The source strength at the sample position was approximately 320 kCi at a dose rate of 10 kGy/h. For dosimetry, 5 mm diameter alanine dosimeters (Bruker Instruments, Rheinstetten, Germany) were used. The dosimeters were calibrated against an international standard set by the International Atomic Energy Agency (Vienna, Austria) to ensure accurate dose measurements. Three plant-derived callus samples (each weighing approximately 10 g) were subjected to gamma irradiation at doses of 0.05, 0.5, or 10 kGy. After irradiation, callus samples were ground and extracted overnight at room temperature using 50 mL of 80% MeOH. The extracts were filtered, and the solvent was evaporated under reduced pressure to yield crude callus extracts.

### 4.3. Measurement of Radical Scavenging Activity

The DPPH radical scavenging activity was assessed using the method described by Blois [28], with minor modifications. A DPPH solution (0.2 mM) was prepared in ethanol (EtOH). Each test extract was prepared at a concentration of 100 μg/mL in 70% EtOH. A 60 μL aliquot of DPPH solution was mixed with 120 μL of each test extract. The mixture was incubated in the dark for 15 min. After incubation, the decrease in absorbance was measured at 517 nm using an ELISA reader.

The ABTS^+^ radical was generated by reacting 5 mL of ABTS^+^ (7 mM in EtOH) with 5 mL of 2.4 mM potassium persulfate (K_2_S_2_O_8_). This mixture was allowed to stand in the dark at 25 °C for 24 h before use. For the ABTS^+^ radical scavenging assay, 100 μL of the ABTS^+^ solution was added to a 96-well plate containing different concentrations of the callus extracts and a positive control (catechin) at 100 μM. The assay mixtures were then stirred for 30 s and incubated for 30 min at 25 °C [29]. The radical scavenging activities of DPPH and ABTS^+^ were calculated using the following equations:

Radical scavenging activity (%) = [1 − (A_2_/A_1_)] × 100

where A_1_ is the absorbance of the control (without the sample) and A_2_ is the absorbance of the test sample. (+)-Catechin was used as a positive control.

### 4.4. High-Performance Liquid Chromatography (HPLC) Analysis

An HPLC analysis was performed using an Agilent HPLC 1200 system (Agilent Technologies, Palo Alto, CA, USA) equipped with a photodiode array detector (PDA; 1200 Infinity series, Agilent Technologies). The system included a series of ZORBAX Eclipse Plus C_18_ columns (4.6 mm i.d. × 150 mm, particle size 3.5 µm; Agilent Technologies). The solvent system was operated in gradient mode. Initially, the mobile phase consisted of 0.1% (HCOOH) in H_2_O. Over a 25 min period, the mobile phase was gradually changed to MeCN. The column temperature was maintained at 40 °C, and the flow rate was set to 1.0 mL/min. The eluting metabolites were detected using a UV detector at wavelengths of 254 and 360 nm.

### 4.5. Ultra-Performance Liquid Chromatography-Quadrupole Time-of-Flight/Mass Spectrometry (UPLC-QTOF/MS) Analysis

For the UPLC-QTOF/MS (Xevo, Waters, Milford, MA, USA) analysis, samples (1 μL) were injected into an Acquity UPLC BEH C_18_ column (2.1 × 100 mm, particle size 1.7 µm; Waters). The column was equilibrated with H_2_O containing 0.1% HCOOH (A) and eluted using a gradient with MeCN (B) at a flow rate of 0.35 mL/min. The elution program was as follows: 0–2.5 min, 85% A; 2.5–7.5 min, 80% A; 7.5–12.5 min, 70% A; 12.5–13.5 min, 100% A; 13.5–20.0 min, 0% A. The eluted metabolites were analyzed by Q-TOF/MS in the electrospray ionization (ESI) positive mode. The capillary voltage was set to 3 kV, and the sampling cone voltage was set to 30 V. The source temperature was maintained at 100 °C, with a desolvation flow rate of 800 L/h and a temperature of 400 °C. TOF/MS data were collected in the 50–1500 *m/z* range at a scan time of 0.2 s. The MS/MS spectra of the extracts were collected in the 50–1000 *m/z* range, using a collision energy ramp from 10 to 30 eV to acquire MS data including *m/z*, retention time (RT), and ion intensity. The data extraction, normalization, and alignment of the MS datasets were performed using the UNIFI software 1.8 package (Waters, Milford, MA, USA). The metabolite identification was performed using the Human Metabolome Database (HMDB; https://www.hmdb.ca, accessed on 10 March 2022), the Metabolite and Chemical Entity (METLIN) database (https://metlin.scripps.edu, accessed on 10 March 2022), ChemSpider (https://www.chemspider.com, accessed on 10 March 2022), literature references, and authentic standards.

### 4.6. Statistical Analysis

The MS datasets processed by the UNIFI software package were subjected to multivariate statistical analysis using SIMCA-P^+^ version 12.0.1 (Umetrics, Umeå, Sweden). Differences among *E. maculata* callus groups were visualized using a partial least squares discriminant analysis (PLS-DA). The quality of the PLS-DA models was evaluated by the goodness of fit measures (R_2_X and R_2_Y) and predictive ability (Q2), and validated by cross-validation with a permutation test (*n* = 200). The metabolites contributing to the differences among the groups were identified based on the variable importance in the projection (VIP) value > 0.7, calculated by a PLS-DA, and with a one-way analysis of variance (ANOVA) with Duncan’s test (*p* < 0.05) using SPSS 17.0 (SPSS Inc., Chicago, IL, USA). The experiments were repeated at least three times to ensure consistent results. The data were expressed as the mean ± standard deviation (SD). Statistical differences between two groups were assessed using an unpaired two-tailed student *t*-test, while differences among multiple groups were analyzed using either one-way or two-way ANOVA with Tukey’s post hoc test. All statistical analyses were performed using GraphPad Prism ver. 8 (San Diego, CA, USA).

## 5. Conclusions

The antioxidant activity of *E. maculata* calli (PC3012) was significantly enhanced by gamma irradiation, as evidenced by the dose-dependent increase in DPPH and ABTS^+^ radical scavenging activities. Detailed HPLC and LC-Q-TOF/MS analyses revealed no newly formed antioxidant compounds but a substantial increase in lysophospholipid content after gamma irradiation, aligned with known plant stress responses. A PLS-DA revealed clear metabolic distinctions between non-irradiated and irradiated calli, as confirmed by robust statistical validation. These findings suggest that gamma irradiation alters specific metabolites, thereby enhancing the bioactive characteristics of calli. This method has potential applications in the development of functional foods and nutraceuticals.

## Figures and Tables

**Table 1:** Metabolites identified in the non-irradiated and irradiated *Euphorbia maculata* calli using UPLC-Q-TOF/MS/MS.

Metabolite No.	RT (min)	Compounds	Exact Mass (*m/z*)	Fragment Ions (*m/z*)	Non- Irradiated	Irradiated
1	0.61	_L_-Arginine	175.12	70	+	+
2	0.65	Pteroside B	381.08	365, 219, 175	+	+
3	0.74	*N*-(1-deoxy-1-fructosyl)isoleucine	294.15	276, 248	+	+
4	0.87	γ-Glutamyl lysine	276.14	258, 230, 212, 147, 86	+	+
5	1.08	Epicatechin 3-glucuronide	467.08	449, 393, 153	+	+
6	1.14	*N*-(1-deoxy-1-fructosyl)phenylalanine	328.14	310, 292, 264	+	+
7	1.19	L-Phenylalanine	166.09	120, 103, 91	+	+
8	1.30	Sinapic acid	225.04	207, 153	+	+
9	1.34	Gallic acid 3-*O*-gallate	323.04	277, 259, 171, 153, 127	+	+
10	1.40	γ-Glutamyglutamic acid	277.04	259, 231, 171	+	−
11	1.59	*N*-(1-deoxy-1-fructosyl)tryptophan	367.15	349, 188, 163	+	+
12	1.71	_L_-Trytophan	205.10	188, 170, 159, 146, 118, 115	+	+
13	1.93	2′,7-Dihydorxy-4′,5′-dimethoxyisoflavone	315.07	297, 153	+	+
14	1.97	Epicatechin 4′-glucuronide	467.08	341, 291, 153	+	+
15	2.12	Quinic acid	193.09	149, 131, 115, 105, 103	+	+
16	2.20	Chlorogenic acid	355.10	193	+	+
17	2.25	Methyl gallate	158.04	153, 141, 123, 97	+	+
18	2.48	Caffeic acid ethyl ester	209.04	177	+	+
19	2.56	Kaempferol 3-rhamnosyl-6″-(4″-acetylrhamnosyl)glucoside	783.07	637, 619	+	+
20	2.60	Acetylpterosin C	277.04	259, 197, 171	+	+
21	2.80	1,2,3,4,6-Pentagallolyglucose	941.09	619	+	+
22	2.99	Phenylalanylglycine	223.06	177, 120	+	+
23	3.48	Leucocyanidin	307.04	275, 247	+	−
24	3.57	Kaempferol 3-rhamnosyl-(6″-acetyl)galactosyl-7-glucoside	783.09	619	+	+
25	3.65	7-Glucosyl-4″glucuronoyl epigallocatechin gallate	797.10	619	+	+
26	3.76	Quercetin 3,7-diglucosyl-4″-galactoside	789.11	771, 303	+	+
27	4.31	Isoquercitrin	465.10	303	+	−
28	4.37	Quercetin 3-coumaroyl-triglucoside	953.10	465, 303	+	−
29	4.45	Quercetin 3-(2‴,6‴-digalloyl)galactoside	769.09	617, 465, 303	+	+
30	4.71	Quercetin 3-(2-galloyl)glucoside	617.12	465, 315, 303, 297, 153	+	+
31	5.13	Isorhamnetin 3-rutinoyl-4′-rhamnoside	771.10	665, 287	+	+
32	5.31	Kaempferol 3-(2″-rhamnosyl-6″-acetyl)galactosyl-7-rhamnoside	785.09	767, 287	+	−
33	5.54	Kaempferol 3-feruloyl-triglucoside	967.12	619, 287, 177	+	−
34	5.69	Isooreientin	449.11	317, 287	+	−
35	14.23	Dehydrophytosphingosine	316.29	298, 280	+	+
36	14.48	LysoPC(18:3)	518.33	184	+	+
37	14.65	LysoPC(18:2)	520.34	184	+	+
38	14.89	LysoPC(16:0)	496.34	184	+	+
39	14.94	LysoPC(18:1)	522.35	184	+	+
40	15.08	Phytosphingsine 1-phosphate	398.23	280	+	−
41	15.20	Methyl phaephoribide B	621.27	531, 487	+	−
42	15.29	Pheophorbide A	593.28	531, 487	+	+
43	15.53	Pheophorbide B	607.29	547	+	+
44	16.06	PC(18:3/18:3)	778.54	184	+	+

+: detected; −: not detected.

## Data Availability

The datasets used and/or analyzed in the current study are available from the corresponding author upon reasonable request.

## Supplementary Materials

The following supporting information can be downloaded from https://www.mdpi.com/article/10.3390/plants13162306/s1, Figure S1: UPLC-QTOF/MS spectra of *Euphorbia maculata* calli; Figure S2: Photographs of the three plant-derived calli; Table S1: Statistical analysis of *Euphorbia maculata* calli.
