# Supplementary material for: Effects of Gamma Irradiation on Changes in Chemical Composition and Antioxidant Activity of Euphorbia maculata Callus
Source: Plants (Basel). 2024 Aug 19;13(16):2306. doi: 10.3390/plants13162306 (PMC11360754; doi:10.3390/plants13162306)
Supplement: Supplementary file 1 [file plants-13-02306-s001.zip › plants-3163714-supplementary.pdf]

Supplementary material

Effect of Gamma Irradiation on Change of Chemical Composition and Antioxidant Activity of *Euphorbia maculata* Callus

Gyeong Han Jeong [1], Shubhpreet Kaur [1], Youngchul Yoo [1], Young Bae Ryu [2], Seo Jun Lee [2], Kwang-Woo Jung [1], Moon-Soo Chung [1], Hyoung-Woo Bai [1,3], Jin-Hong Kim [1,3], Sungbeom Lee [1,3], Tae Hoon Kim [4], Byung Yeoup Chung [1], Seung Sik Lee [1,3,*]

[1] Advanced Radiation Technology Institute (ARTI), Korea Atomic Energy Research Institute (KAERI), Jeongeup 56212, Republic of Korea; jkh4598@kaeri.re.kr (G.H.J.); shubhpreetk49@gmail.com (S.K.); yooyc84@kakao.com (Y.Y.); kwjung@kaeri.re.kr (K.W.J.); mschung@kaeri.re.kr (M.S.C.); hbai@kaeri.re.kr (H.W.B.); jhongkim@kaeri.re.kr (J.H.K.); sungbeom@kaeri.re.kr (S.L.); bychung@kaeri.re.kr (B.Y.C.)

[2] Functional Biomaterial Research Center, Korea Research Institute of Bioscience and Biotechnology (KRIBB), Jeongeup 56212, Republic of Korea; ybryu@kribb.re.kr (Y.-B.R.); lji613@kribb.re.kr (S.J.L.)

[3] Department of Radiation Science, University of Science and Technology (UST), Daejeon 34113, Republic of Korea

[4] Department of Food Science and Biotechnology, Daegu University, Gyeongsan 38453, Republic of Korea; skyey7@daegu.ac.kr (T.H.K.)

* Correspondence: sslee@kaeri.re.kr; Tel.: +82-63-580-3332

Contents

Figure S1. UPLC-QTOF/MS spectrum of *Euphorbia maculate* callus.

1. L-Arginine
2. Pteroside B
3. *N*-(1-deoxy-1-fructosyl)isoleucine
4. γ-Glutamyl lysine
5. Epicatechin 3-glucuronide
6. *N*-(1-deoxy-1-fructosyl)phenylalanine
7. L-Phenylalanine
8. Sinapic acid
9. Gallic acid 3-*O*-gallate
10. γ-Glutamyglutamic acid
11. *N*-(1-deoxy-1-fructosyl)tryptophan
12. L-Trytophan
13. 2',7-Dihydorxy-4',5'-dimethoxyisoflavone
14. Epicatechin 4'-glucuronide
15. Quinic acid
16. Chlorogenic acid
17. Methyl gallate
18. Caffeic acid ethyl ester
19. Kaempferol 3-rhamnosyl-6''-(4''-acetylrhamnosyl)glucoside
20. Acetylpterosin C
21. 1,2,3,4,6-Pentagallolyglucose
22. Phenylalanylglycine
23. Leucocyanidin
24. Kaempferol 3-rhamnosyl-(6''-acetyl)galactosyl-7-glucoside
25. 7-Glucosyl-4''glucuronoyl epigallocatechin gallate
26. Quercetin 3,7-diglucosyl-4'-galactoside
27. Isoquercitrin
28. Quercetin 3-coumaroyl-triglucoside
29. Quercetin 3-(2''',6'''-digalloyl)galactoside
30. Quercetin 3-(2-galloyl)glucoside
31. Isorhamnetin 3-rutinoyl-4'-rhamnoside
32. Kaempferol 3-(2''-rhamnosyl-6''-acetyl)galactosyl-7-rhamnoside
33. Kaempferol 3-feruloyl-triglucoside
34. Isooreientin
35. Dehydrophytosphingosine
36. LysoPC(18:3)
37. LysoPC(18:2)
38. LysoPC(16:0)
39. LysoPC(18:1)
40. Phytosphingsine 1-phosphate
41. Methyl phaephoribide B
42. Pheophorbide A
43. Pheophorbide B
44. PC(18:3/18:3)

Figure S2. Photographs of the three plant-derived calli

Table S1. Statistical analysis of *Euphorbia maculate* callus.

Figure S1. UPLC-QTOF/MS spectrum of *Euphorbia maculate* callus.

1. 0.61 min: L-Arginine (175.12 = 70)

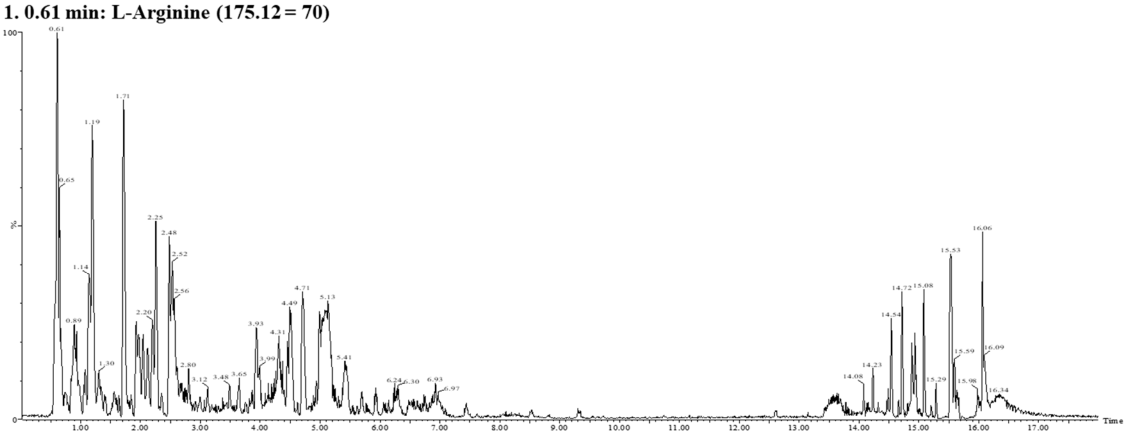

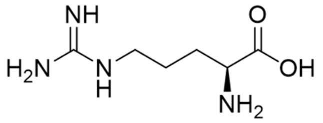

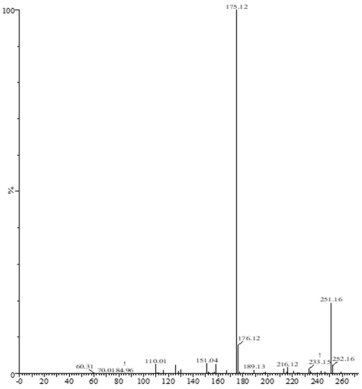

2. 0.65 min: Pteroside B (381.08 = 365, 219, 175)

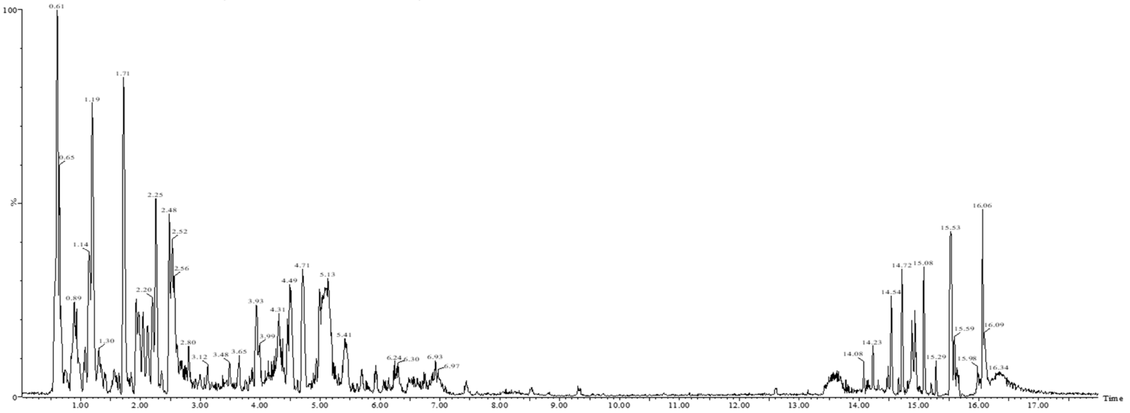

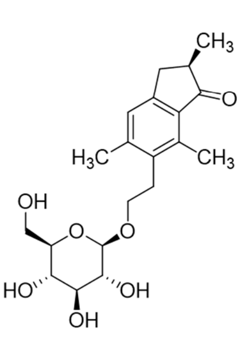

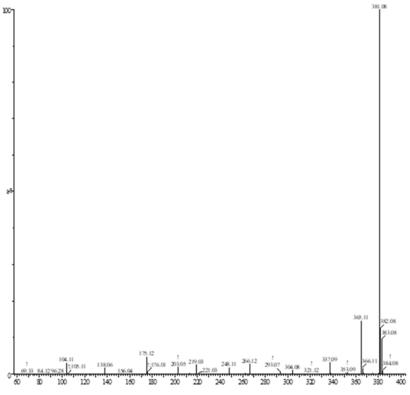

3. 0.74 min: *N*-(1-Deoxy-1-fructosyl)isoleucine (294.15 = 276, 248)

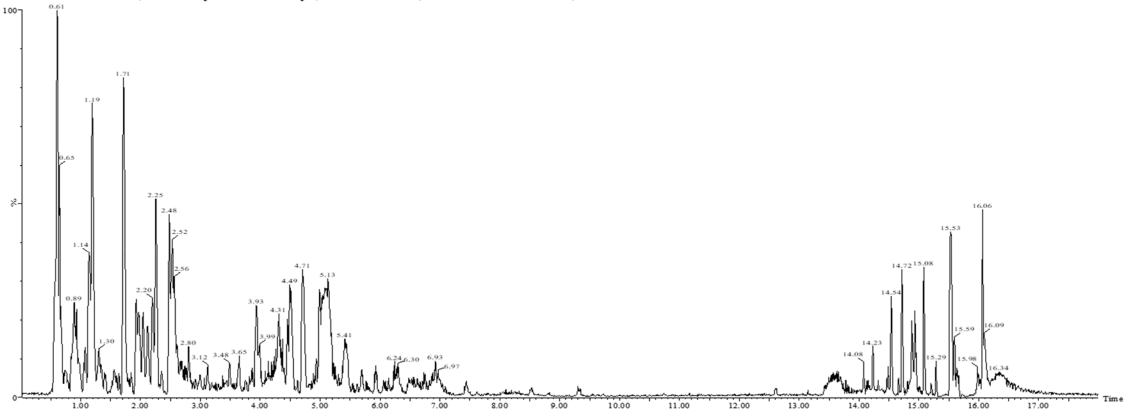

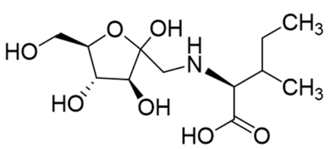

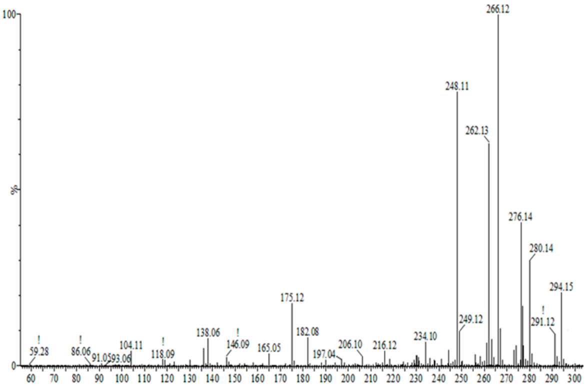

4. 0.87 min: γ-Glutamyl lysine (276.14 = 258, 230, 212, 147, 86)

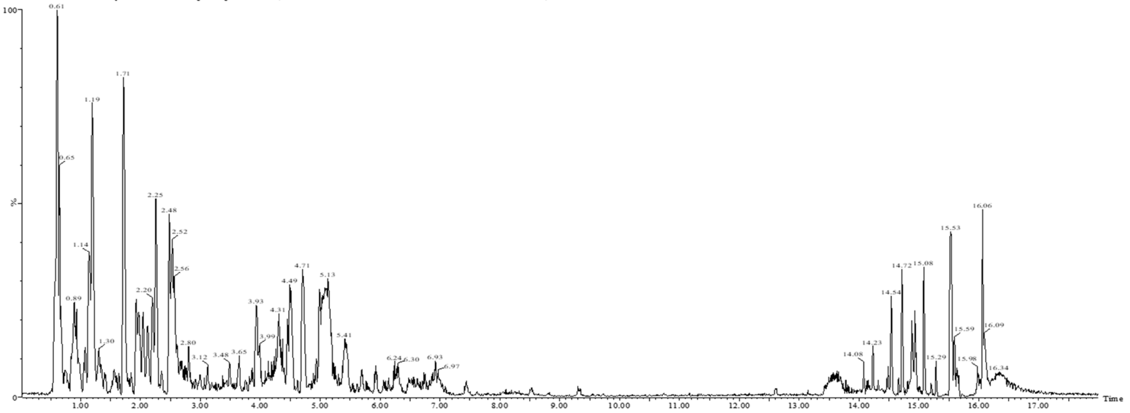

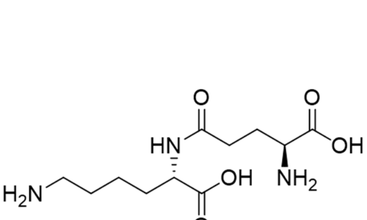

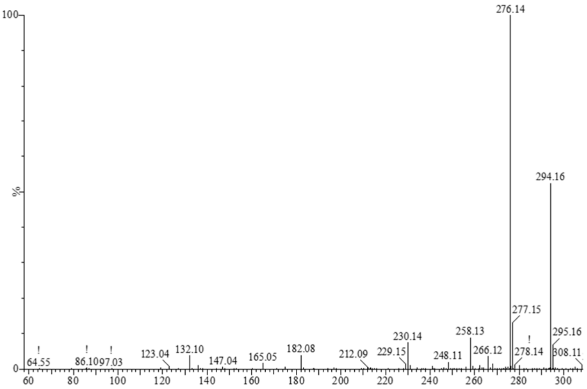

5. 1.08 min: Epicatechin 3-glucuronide (467.08 = 449, 393, 153)

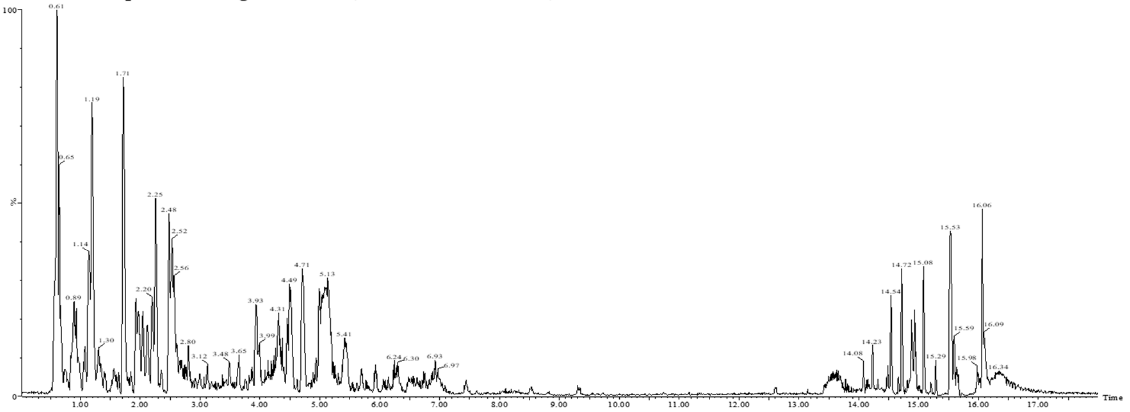

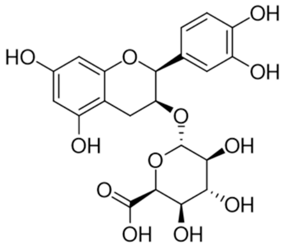

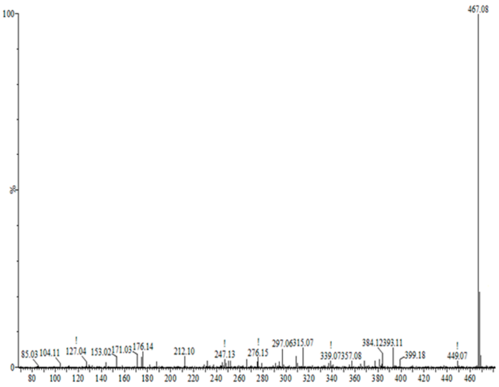

6. 1.14 min: N-(1-Deoxy-1-fructosyl)phenylalanine (328.14 = 310, 292, 264)

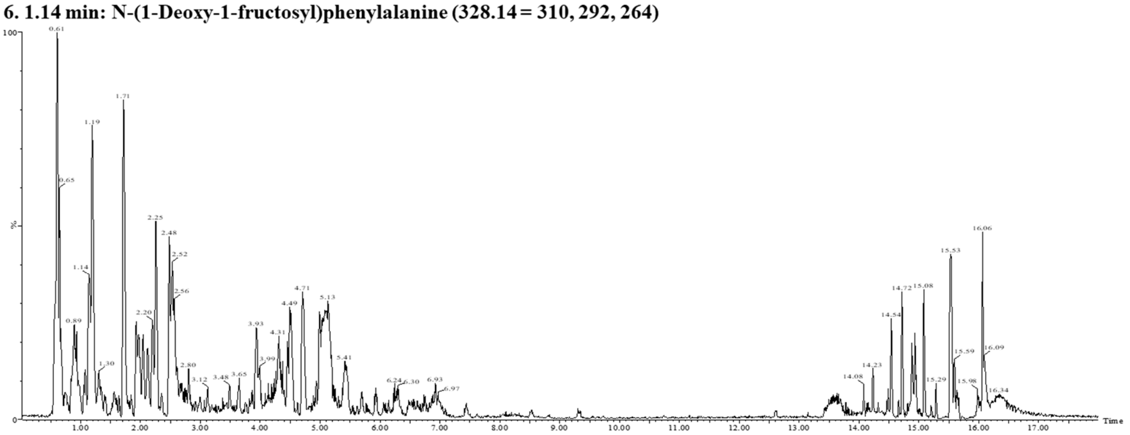

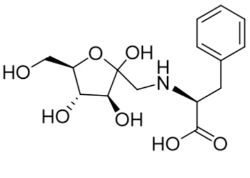

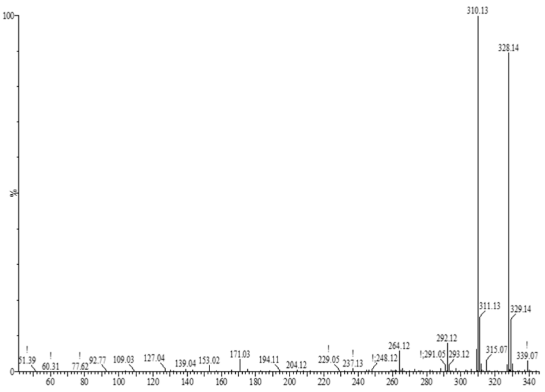

7. 1.19 min: $_{L}$-Phenylalanine (166.09 = 120, 103, 91)

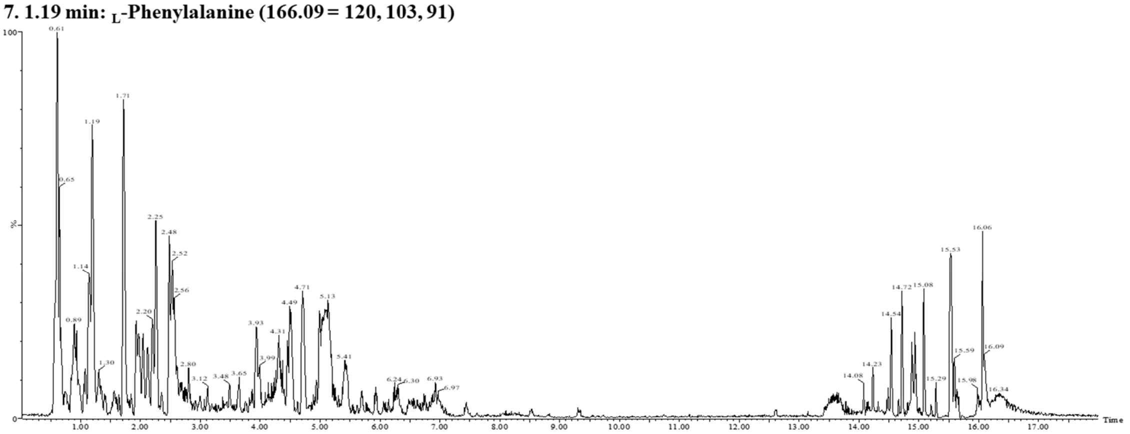

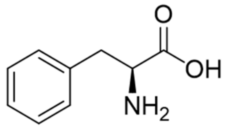

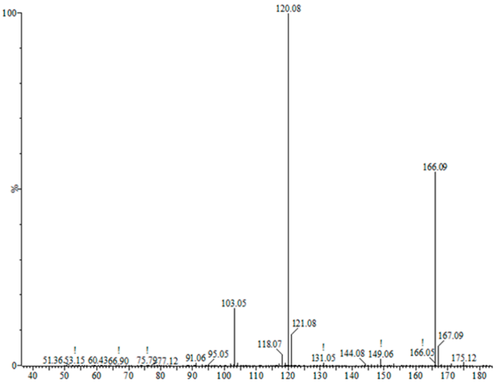

9. 1.34 min: Gallic acid 3-*O*-gallate (323.04 = 277, 259, 171, 153, 127)

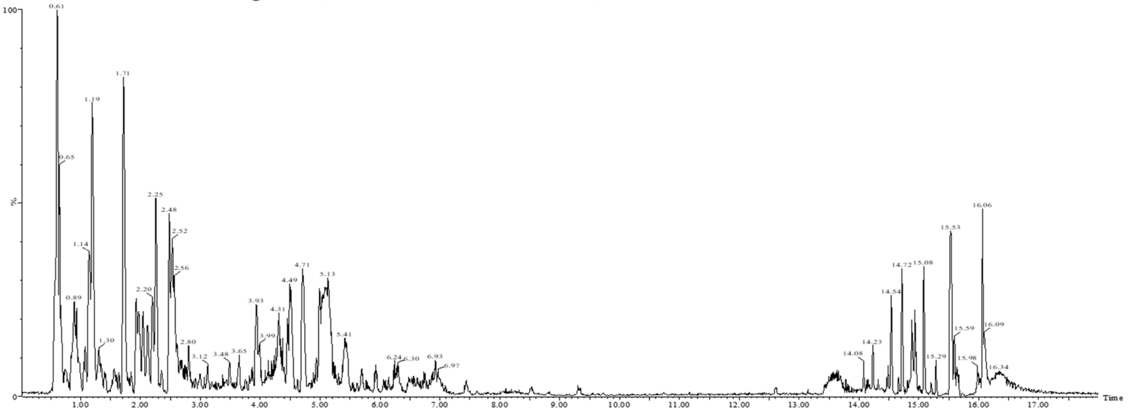

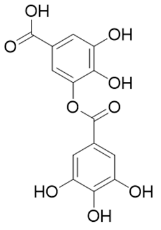

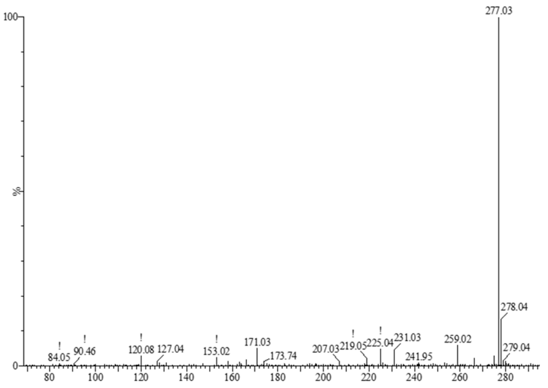

8. 1.30 min: Sinapic acid (225.04 = 207, 153)

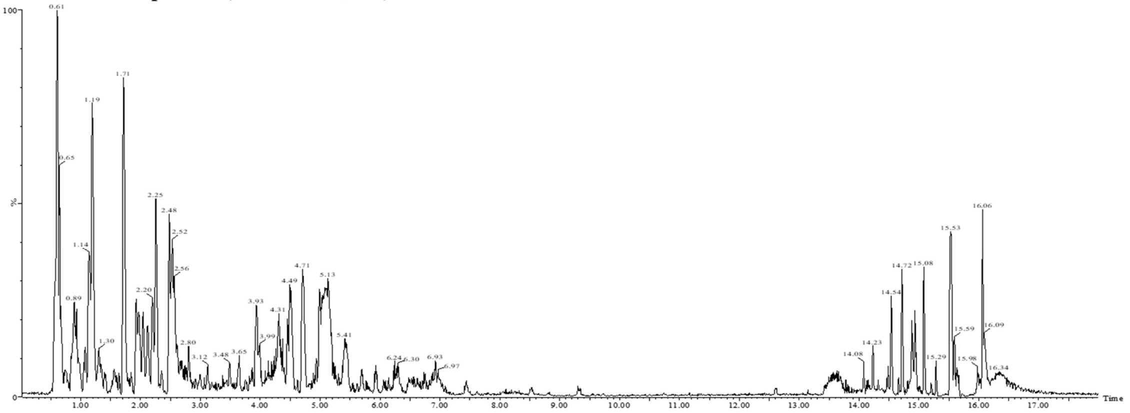

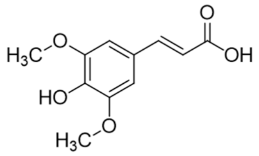

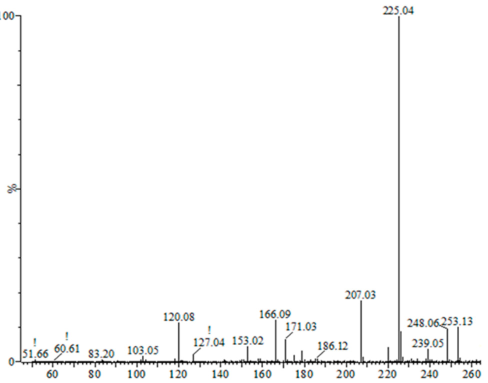

10. 1.40 min: γ-Glutamylglutamic acid (277.04 = 259, 231, 171)

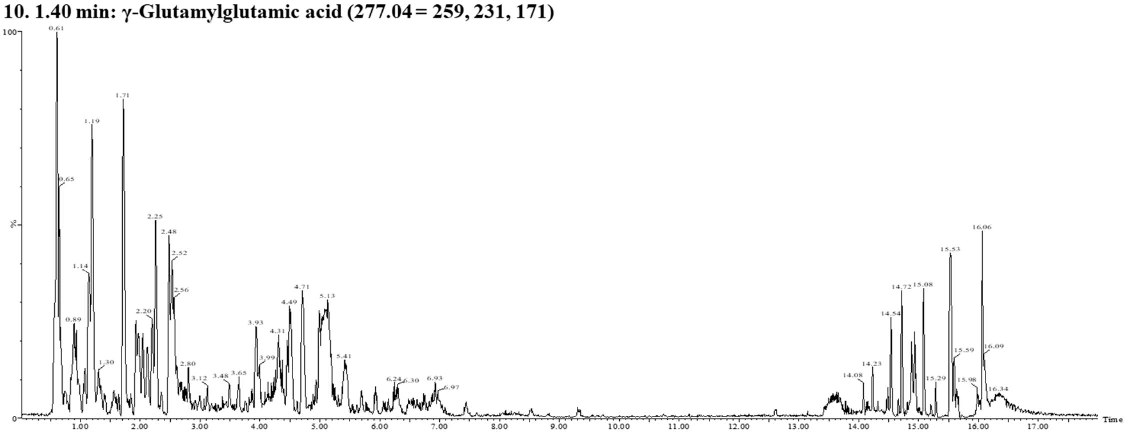

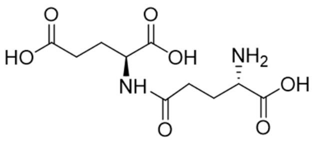

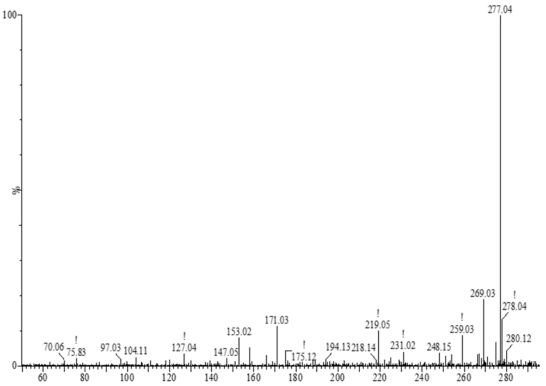

11. 1.59 min: *N*-(1-Deoxy-1-fructosyl)tryptophan (367.15 = 349, 188, 163)

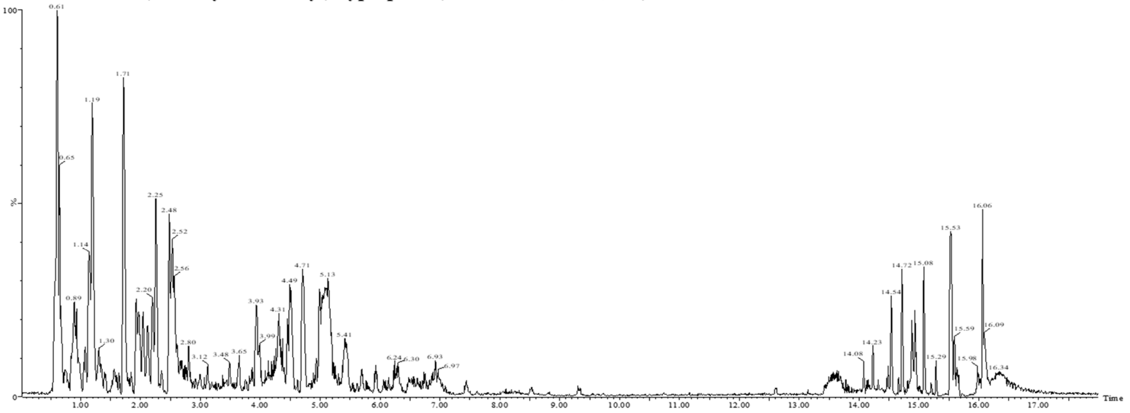

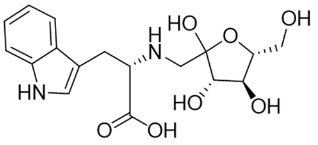

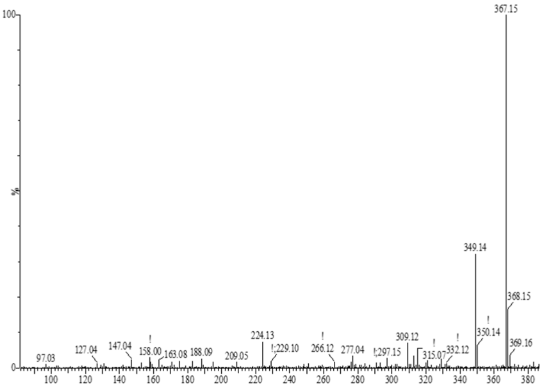

12. 1.71 min: $_{L}$-Tryptophan (205.10 = 188, 170, 159, 146, 118, 115)

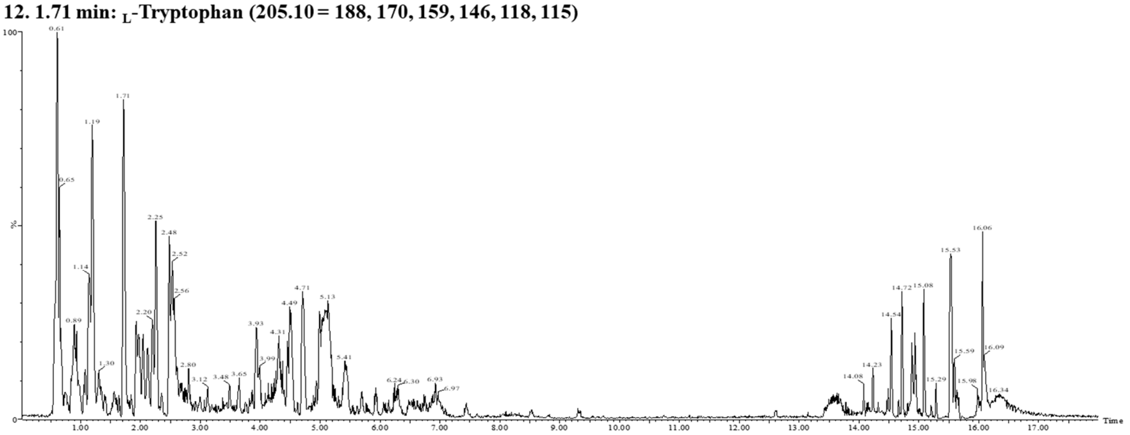

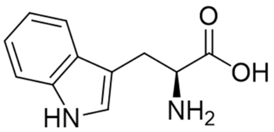

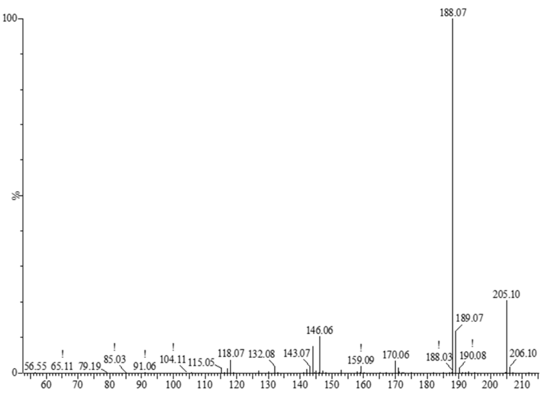

13. 1.93 min: 2',7-Dihydroxy-4',5'-dimethoxyisoflavone (315.07 = 297, 153)

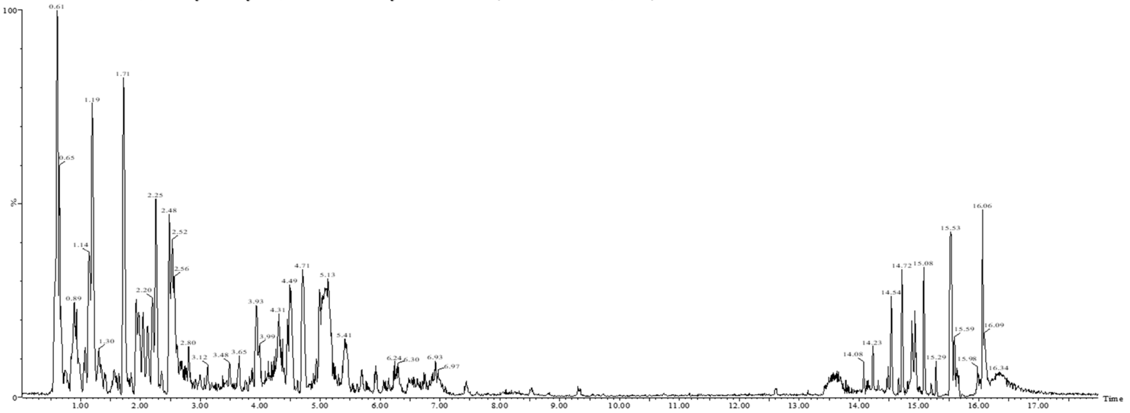

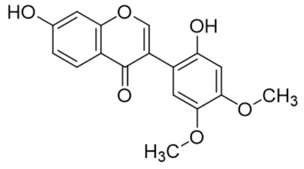

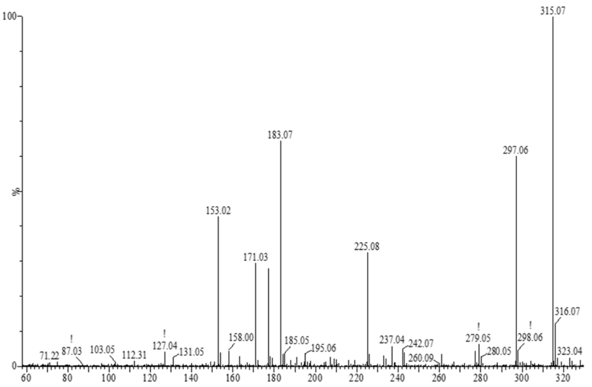

14. 1.97 min: Epicatechin 4'-glucuronide (467.08 = 341, 291, 153)

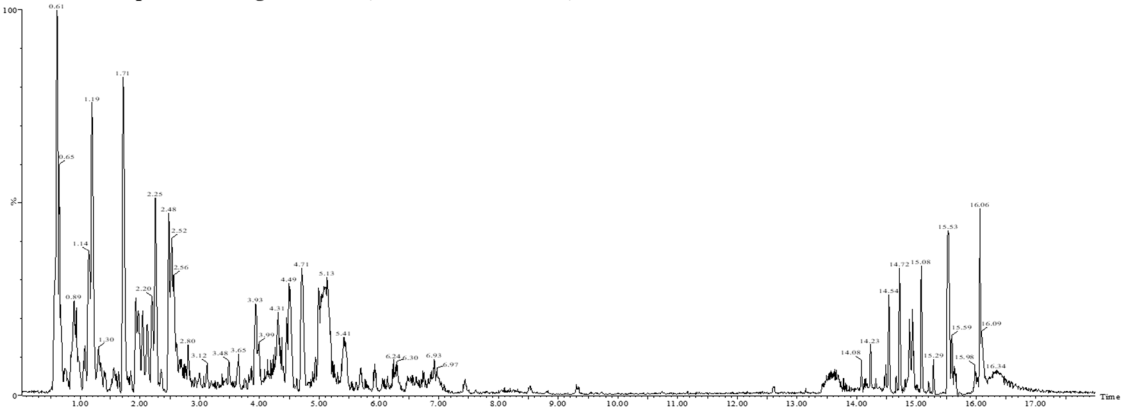

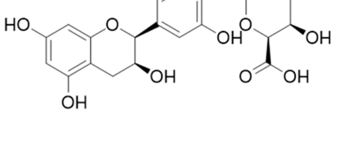

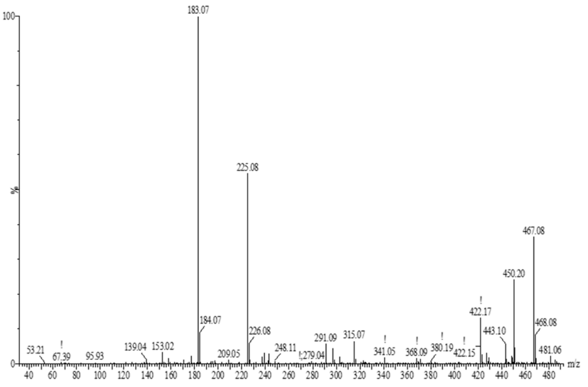

15. 2.12 min: Quinic acid (193.09 = 149, 131, 115, 105, 103)

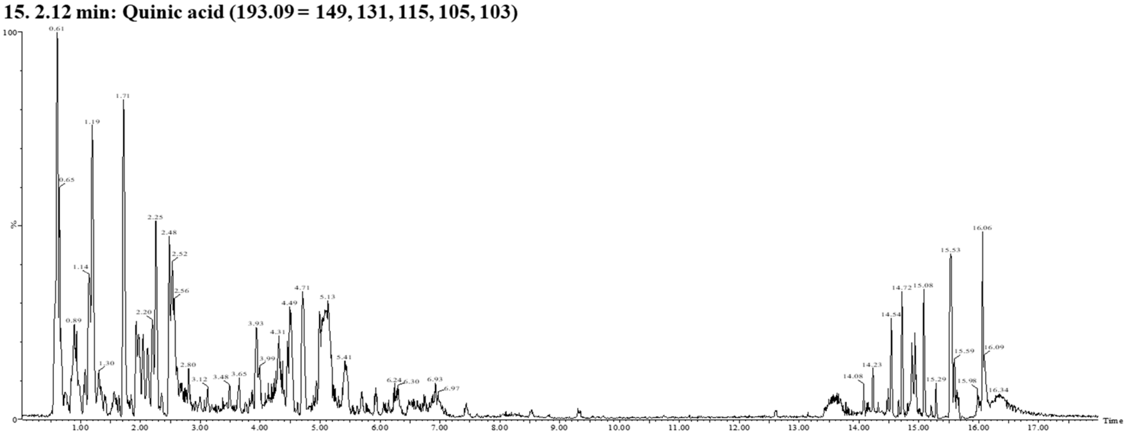

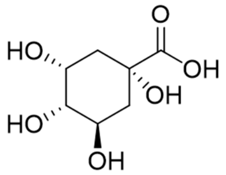

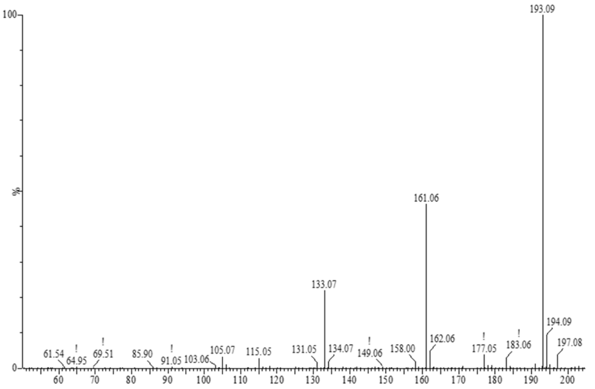

16. 2.20 min: Chlorogenic acid (355.10 = 193)

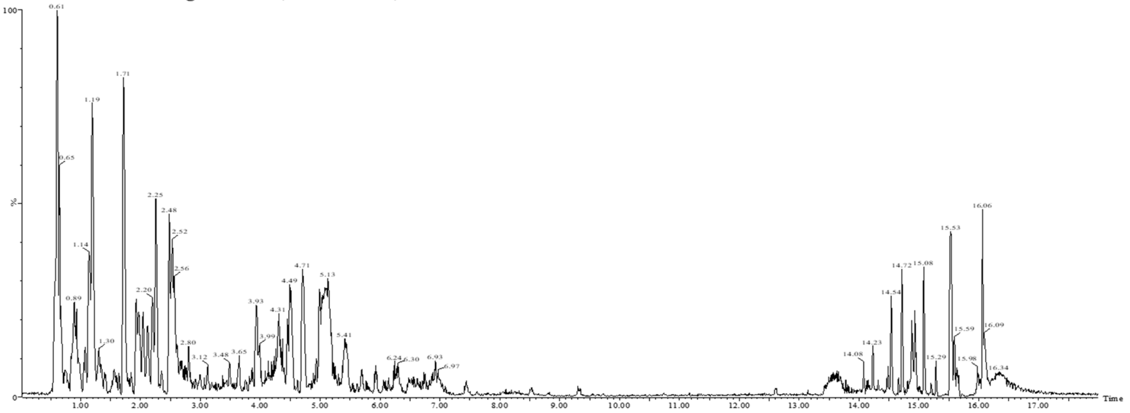

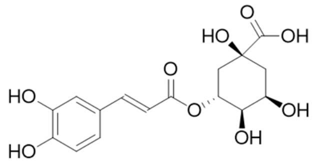

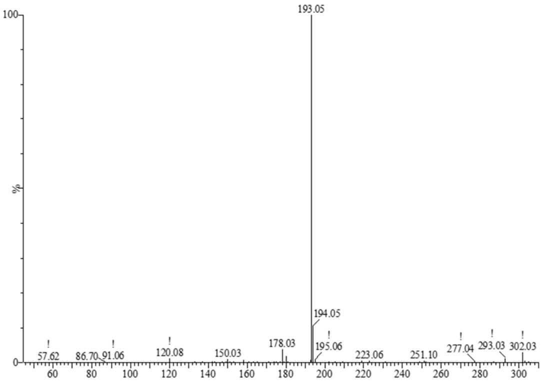

17. 2.25 min: Methyl gallate (185.04 = 153, 141, 123, 97)

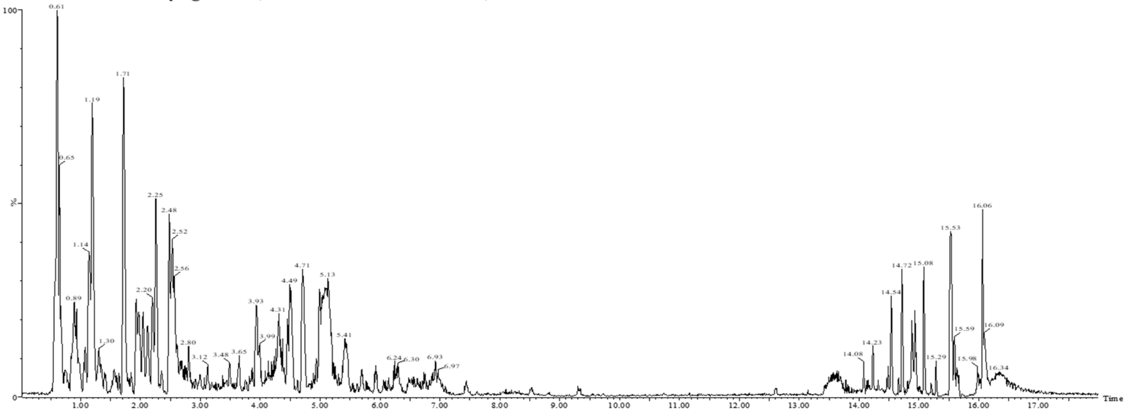

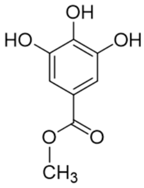

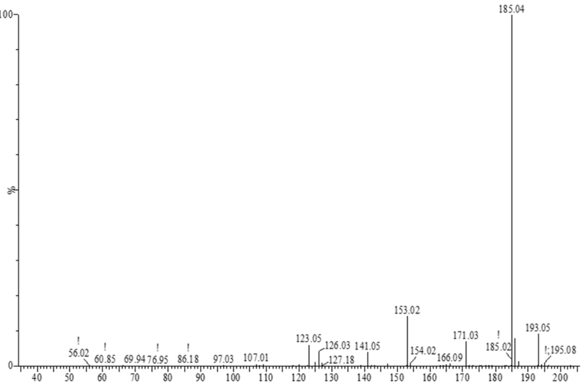

16. 2.48 min: Caffeic acid ethyl ester (209.04 = 177)

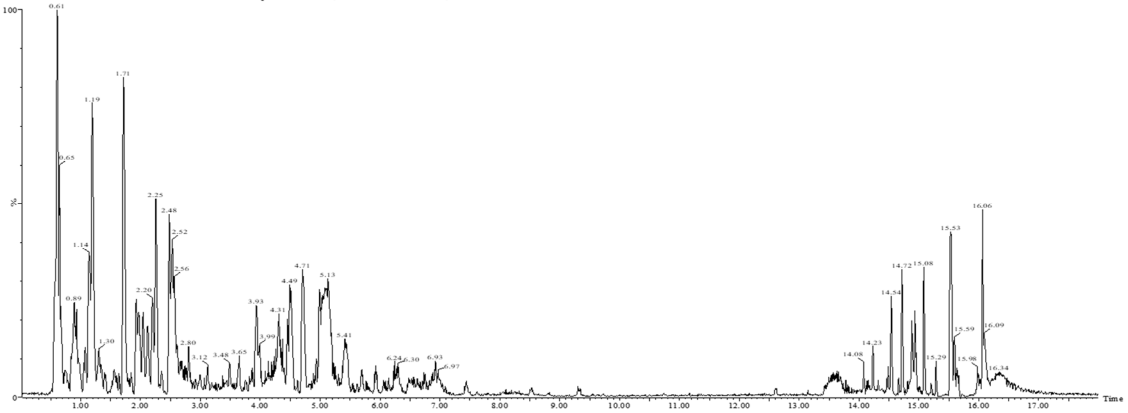

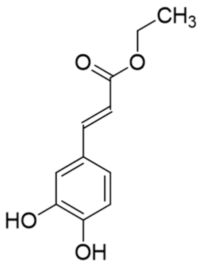

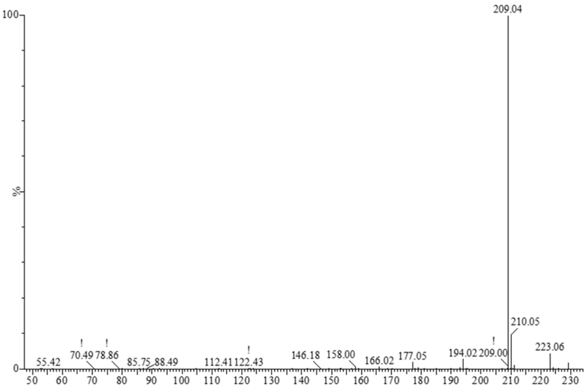

19. 2.56 min: Kaempferol 3-rhamnosyl-6''-(4''-acetylrhamnosyl)glucoside (783.07 = 637, 619)

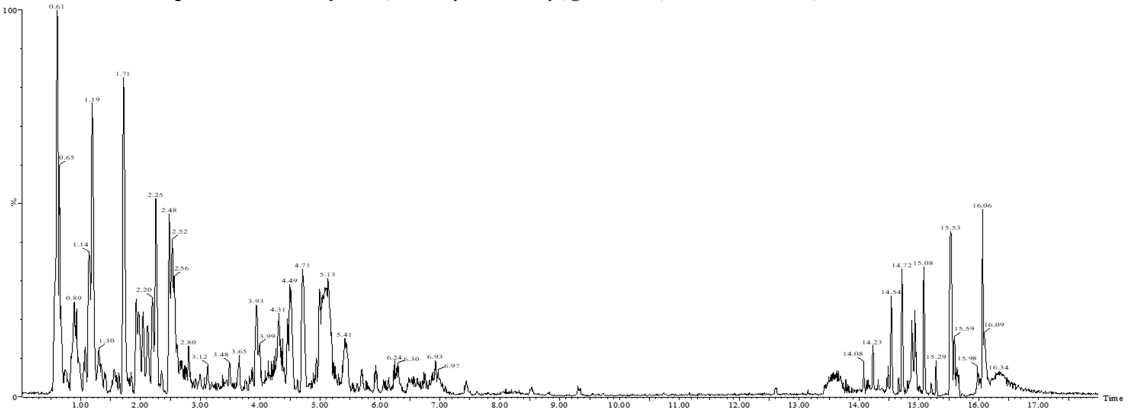

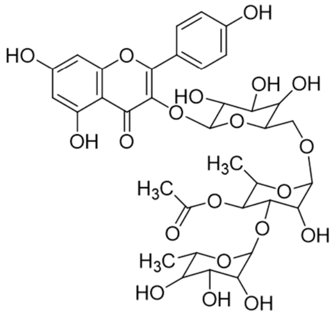

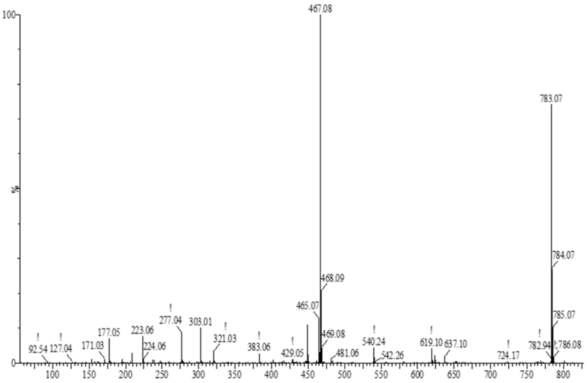

21. 2.80 min: 1,2,3,4,6-pentagalloylglucose (941.09 = 619)

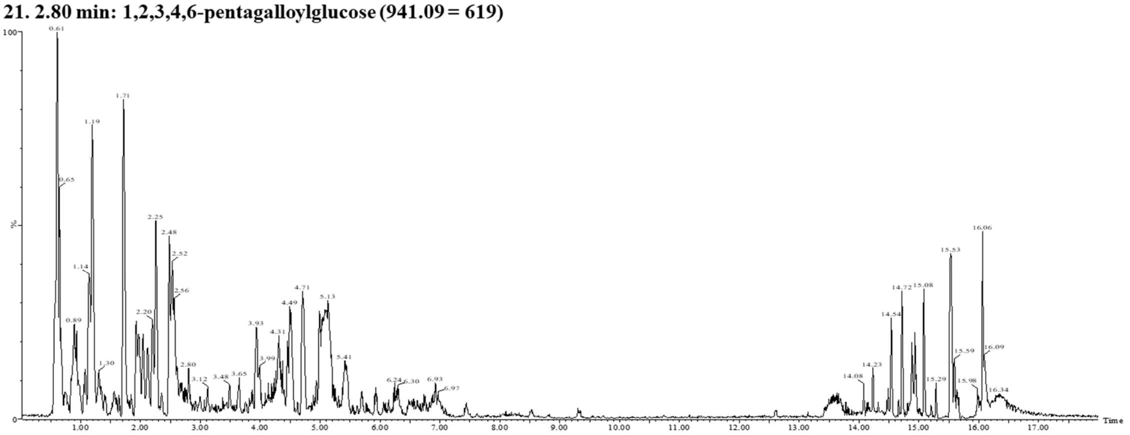

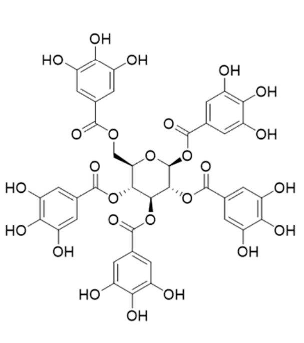

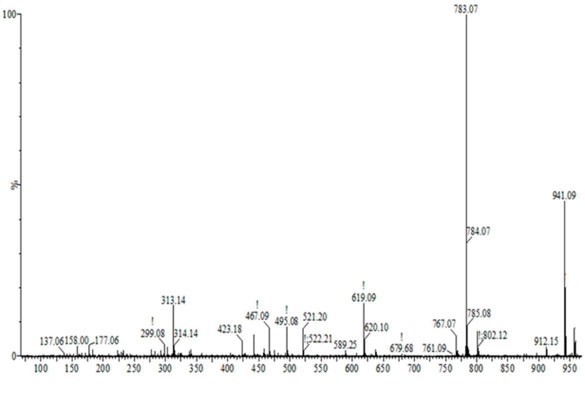

20. 2.60 min: Acetylpterosin C (277.04 = 259, 197, 171)

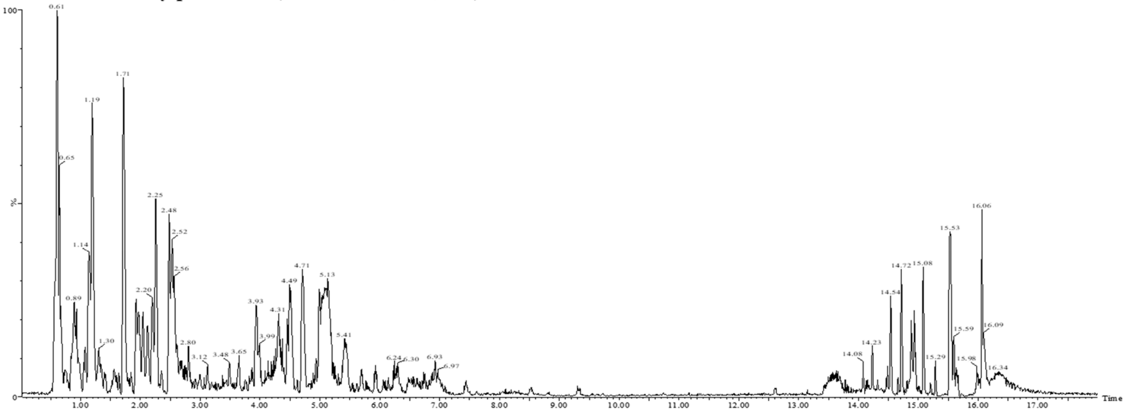

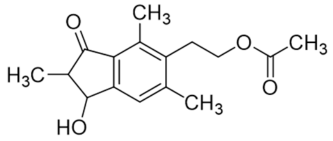

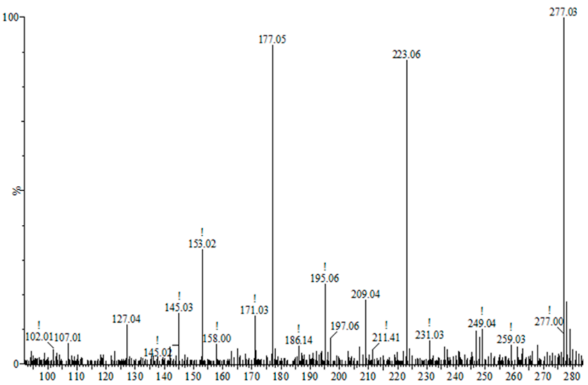

22. 2.99 min: Phenylalanylglycine (223.06 = 177, 120)

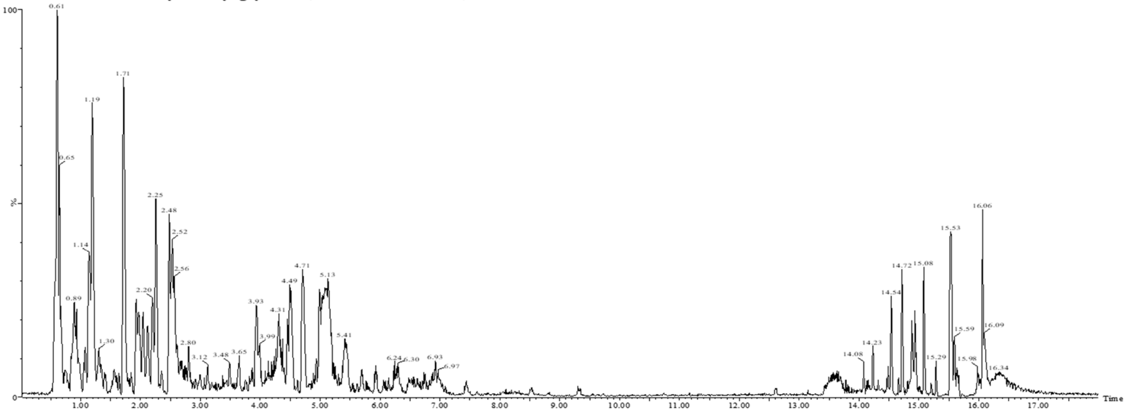

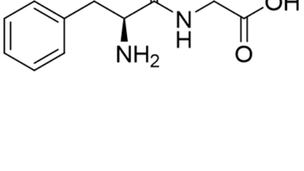

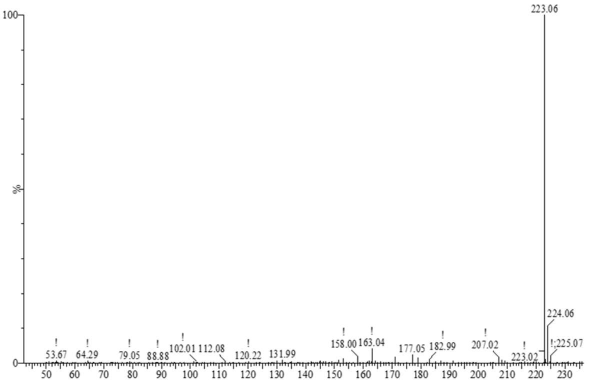

23. 3.48 min: Leucocyanidin (307.04 = 275, 247)

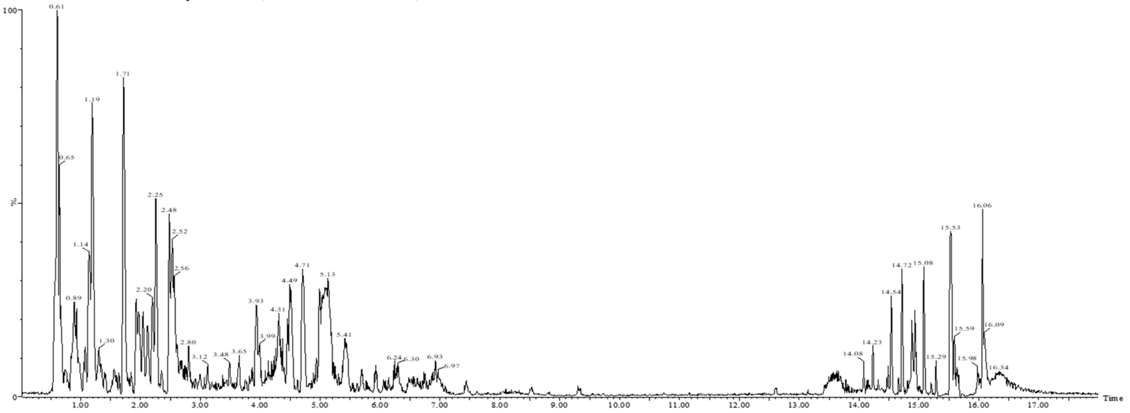

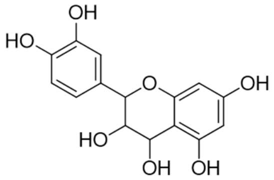

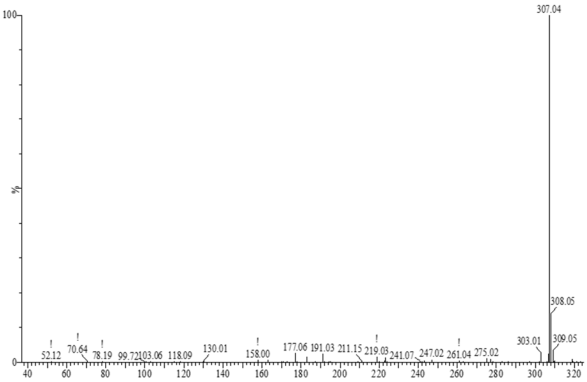

24. 3.57 min: Kaempferol 3-rhamnosyl-(6''-acetyl)galactosyl-7-glucoside (783.09 = 619)

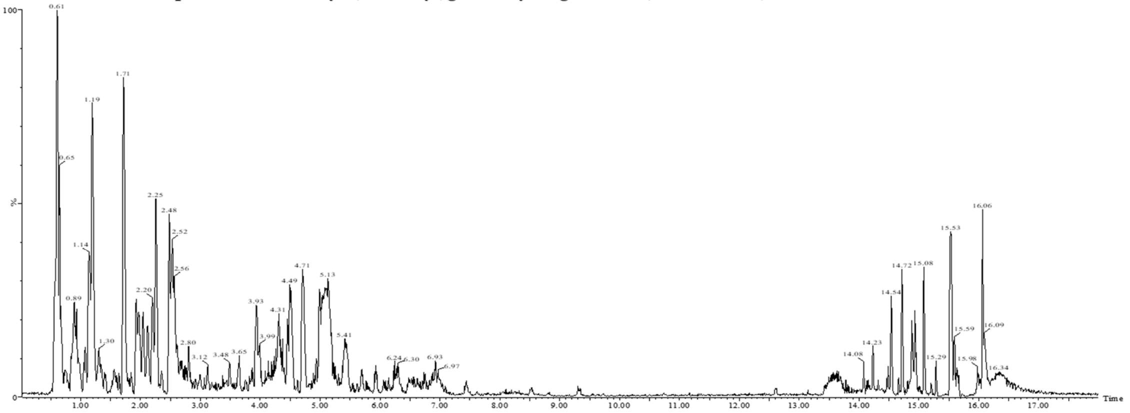

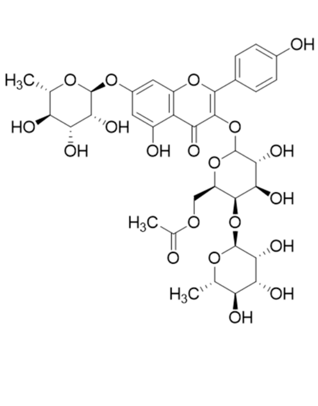

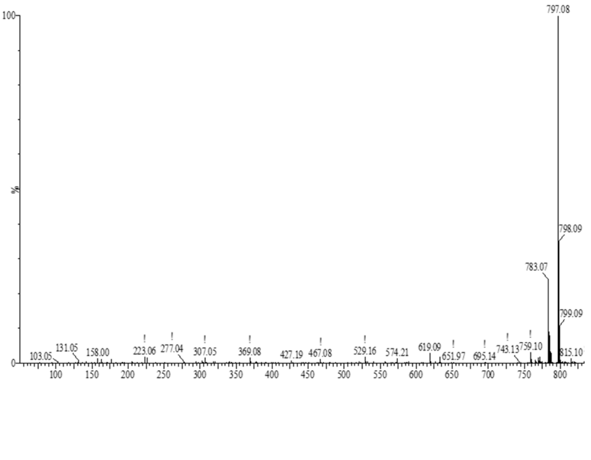

25. 3.65 min: 7-Glucosyl-4''glucuronoyl epigallocatechin gallate (797.10 = 619)

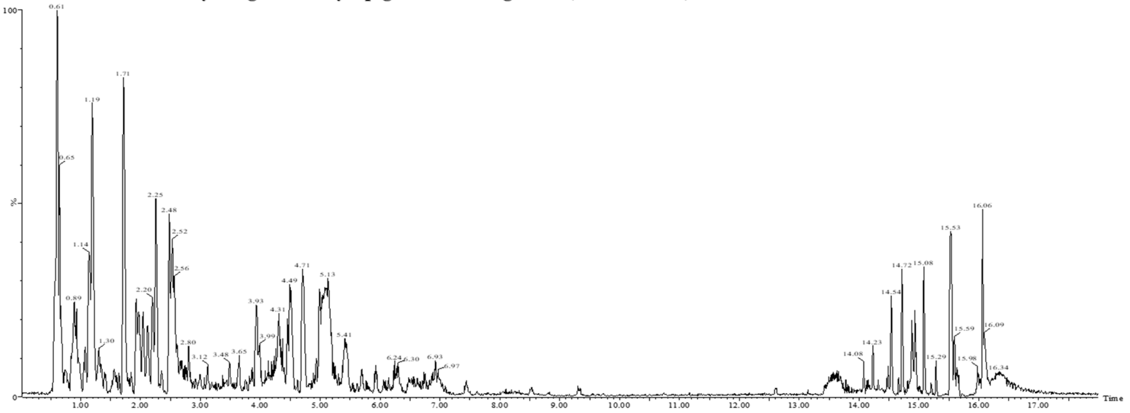

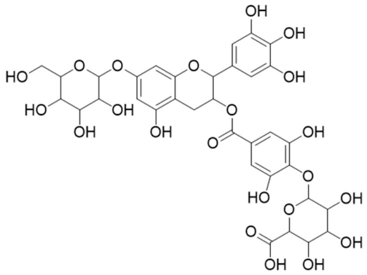

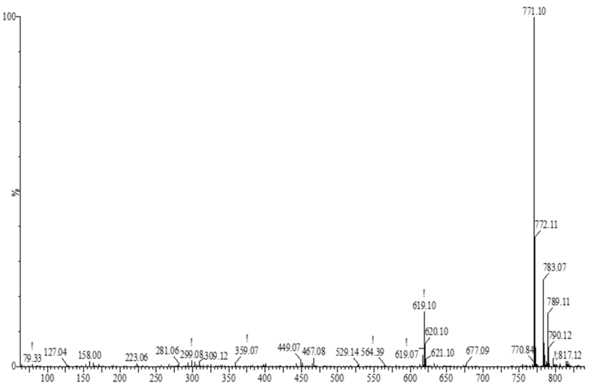

26. 3.76 min: Quercetin 3,7-diglucosyl-4''-galactoside (789.11 = 771, 303)

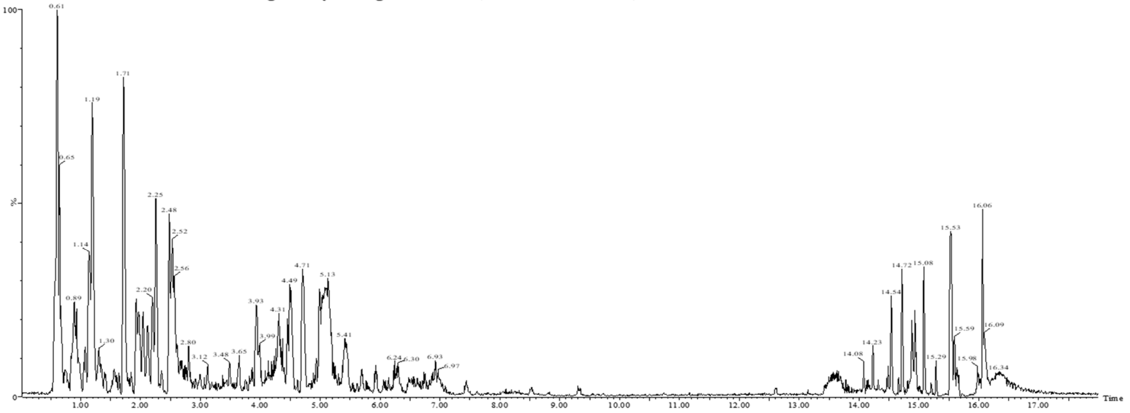

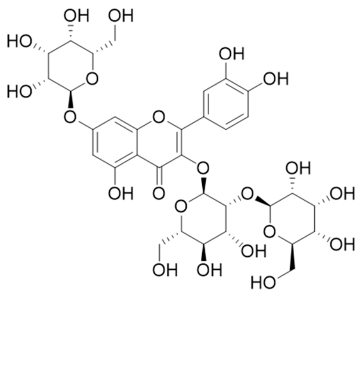

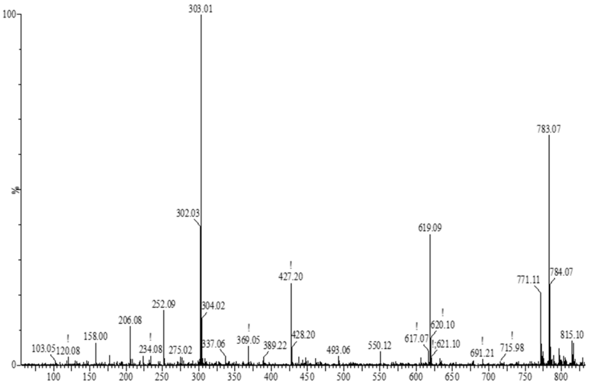

27. 4.31 min: Isoquercitrin (465.10 = 303)

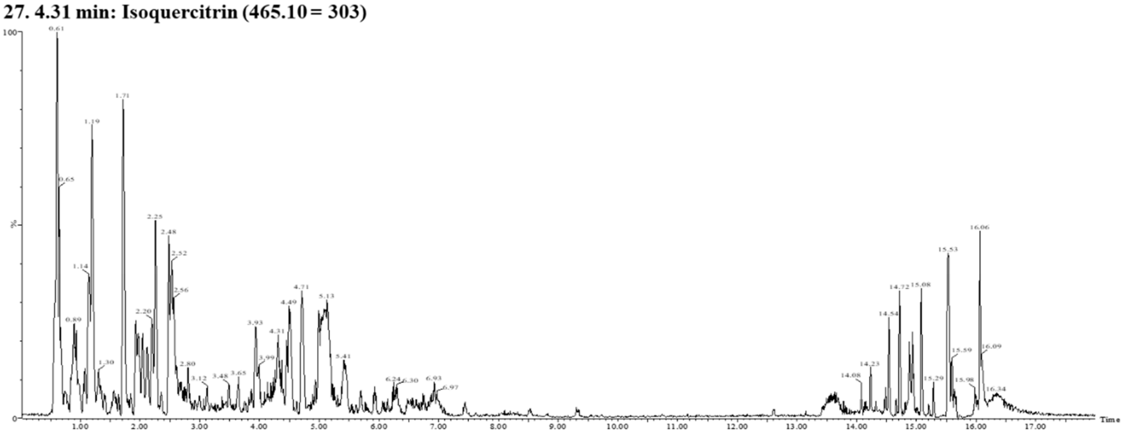

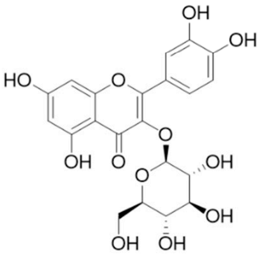

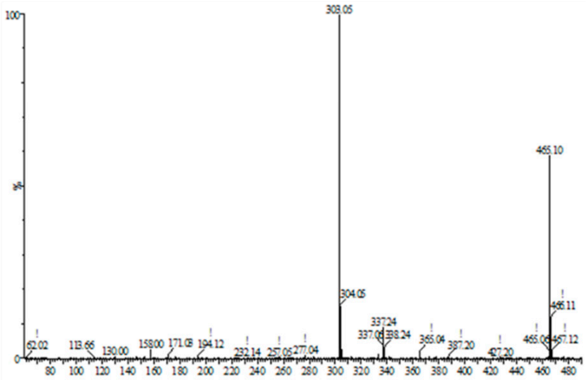

28. 4.37 min: Quercetin 3-coumaroyl-triglucoside (953.10 = 465, 303)

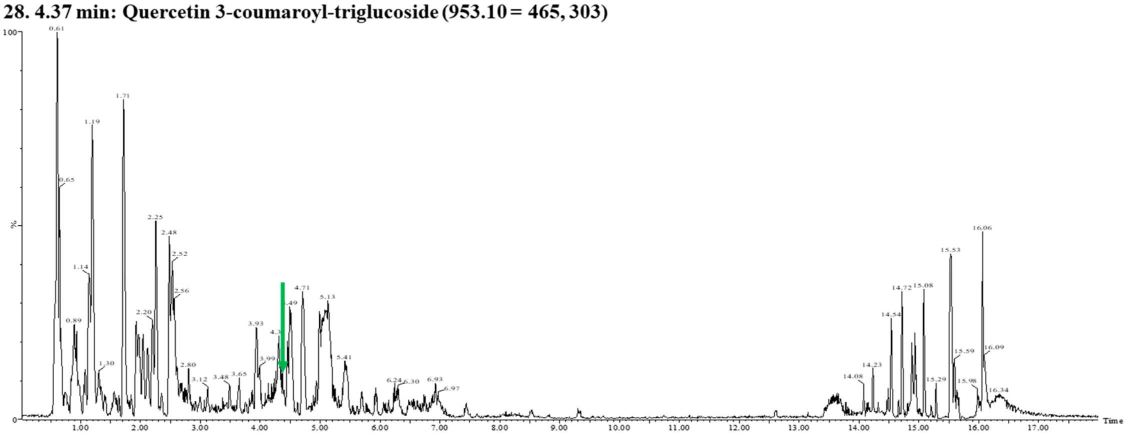

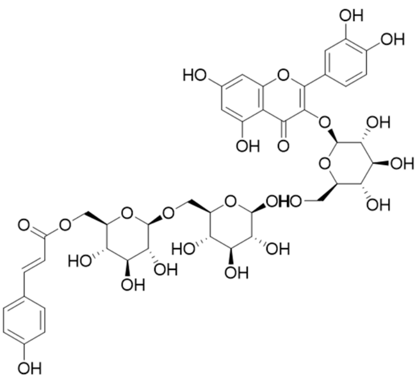

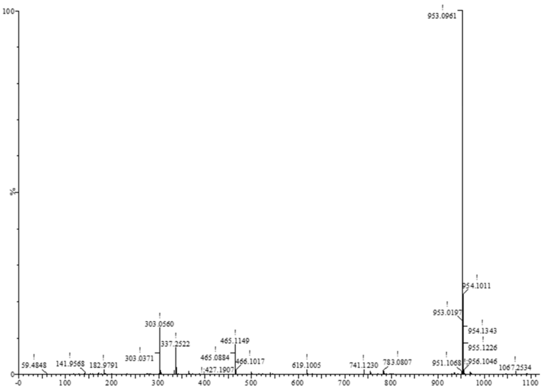

29. 4.45 min: Quercetin 3-(2'',6''-digalloylgalactoside) (769.09 = 617, 465, 303)

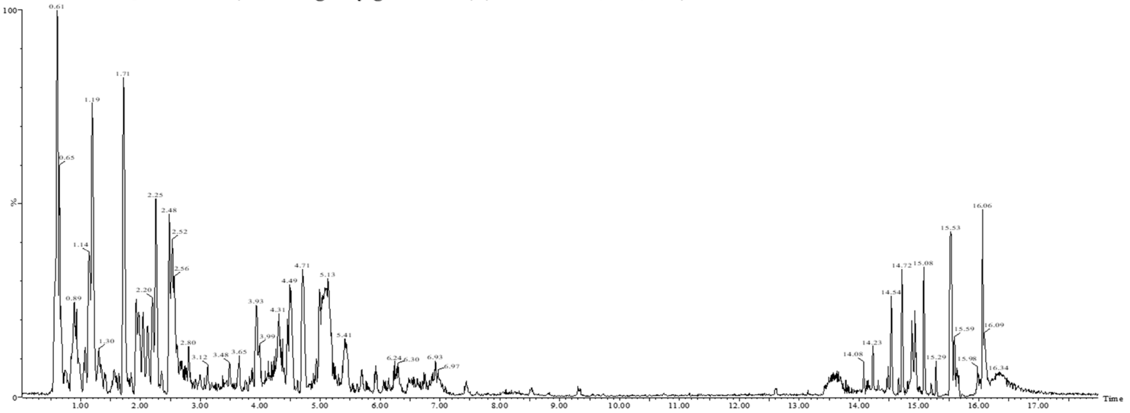

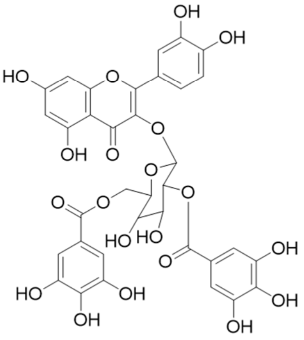

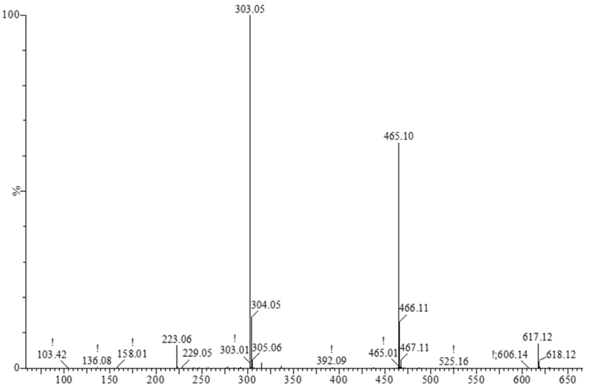

30. 4.71 min: Quercetin 3-(2-galloylglucoside) (617.12 = 465, 315, 303, 297, 153)

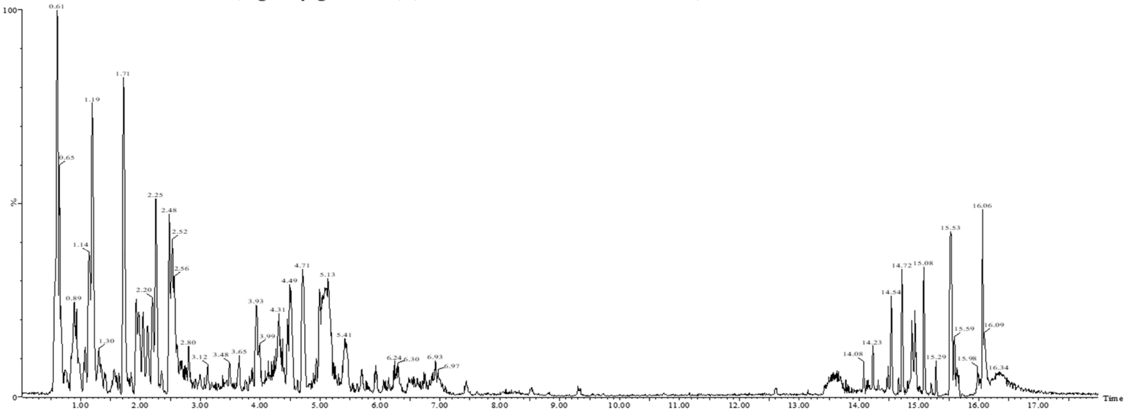

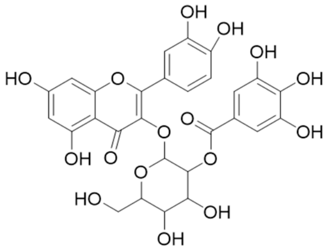

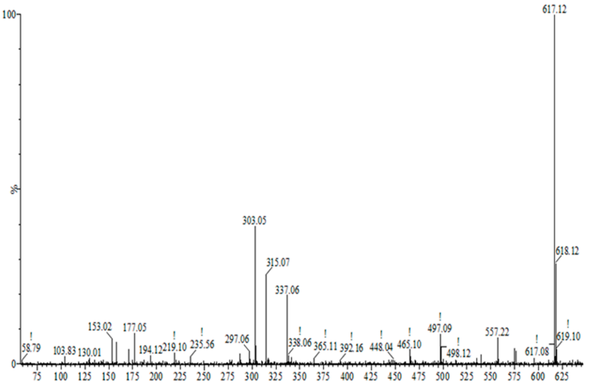

31. 5.13 min: Isorhamnetin 3-rutinoside 4'-rhamnoside (771.10 = 665, 287)

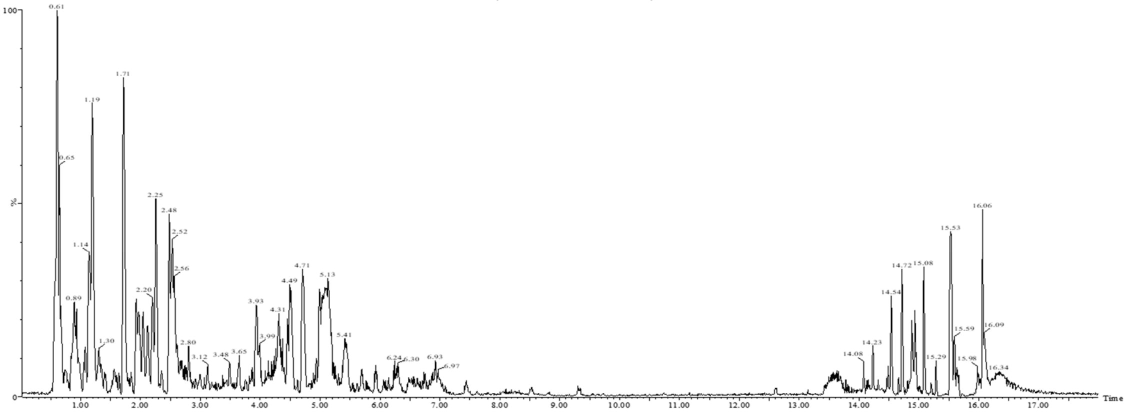

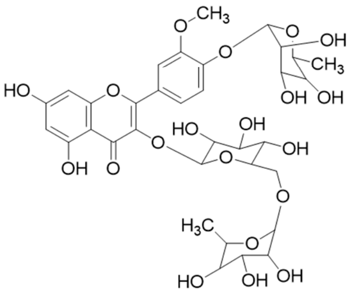

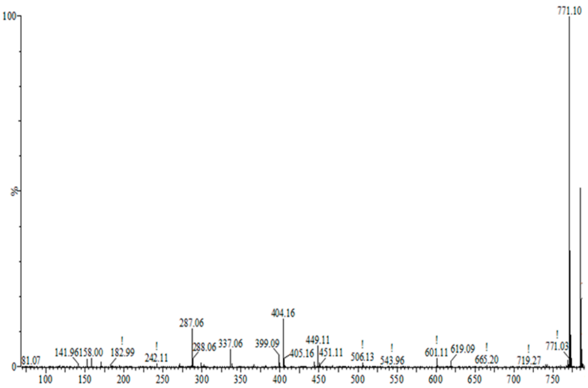

32. 5.31 min: Kaempferol 3-(2''-rhamnosyl-6''-acetyl)galactosyl-7-rhamnoside (785.09 = 767, 287)

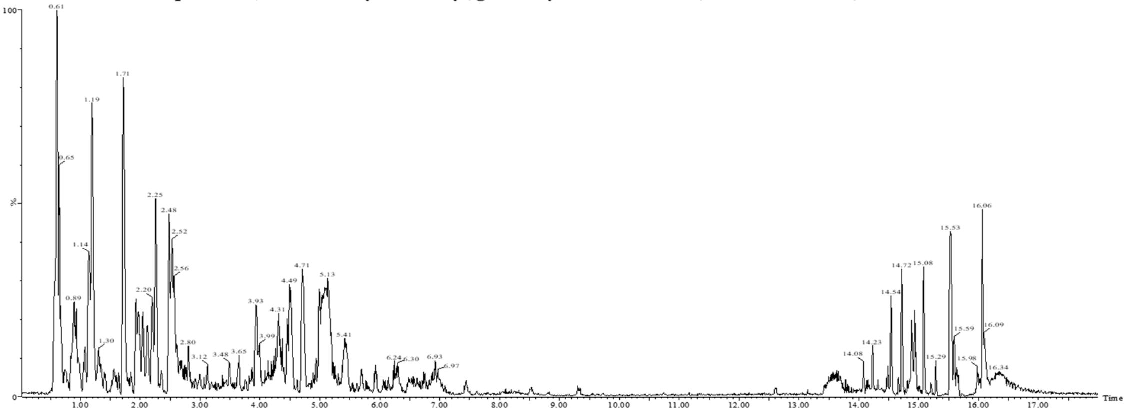

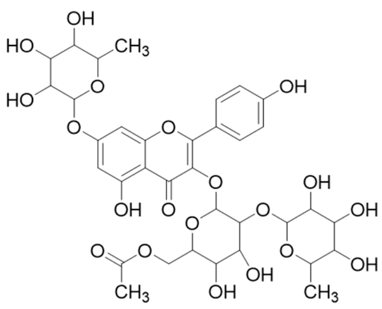

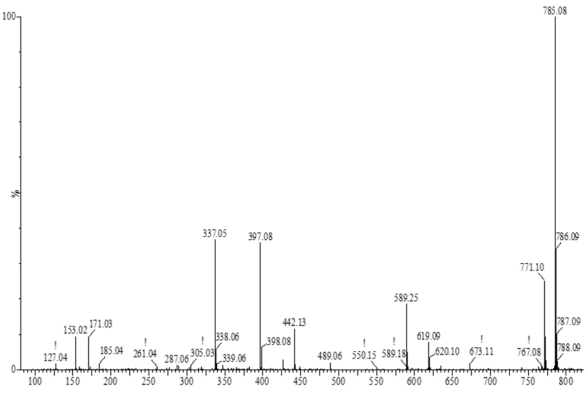

33. 5.54 min: Kaempferol 3-feruloyl-triglucoside (967.12 = 619, 287, 177)

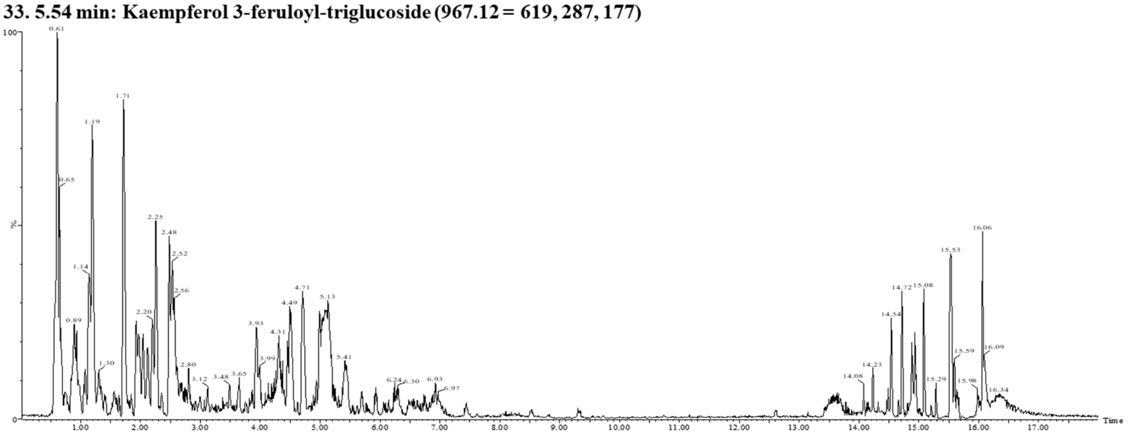

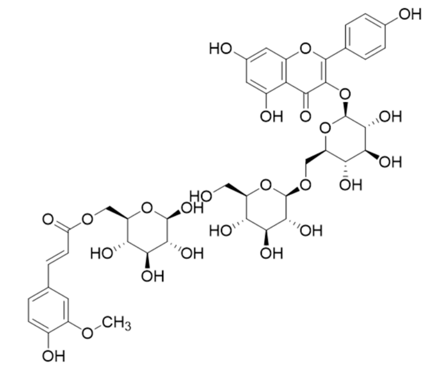

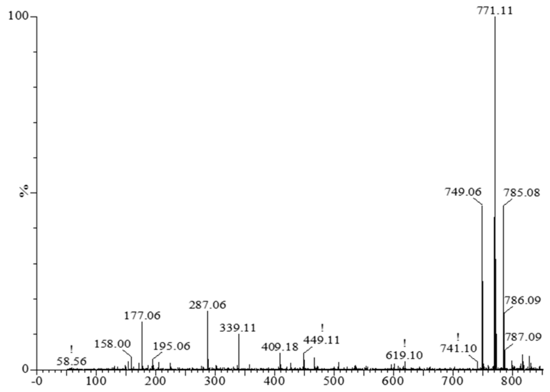

34. 5.69 min: Isoorientin (449.11 = 317, 287)

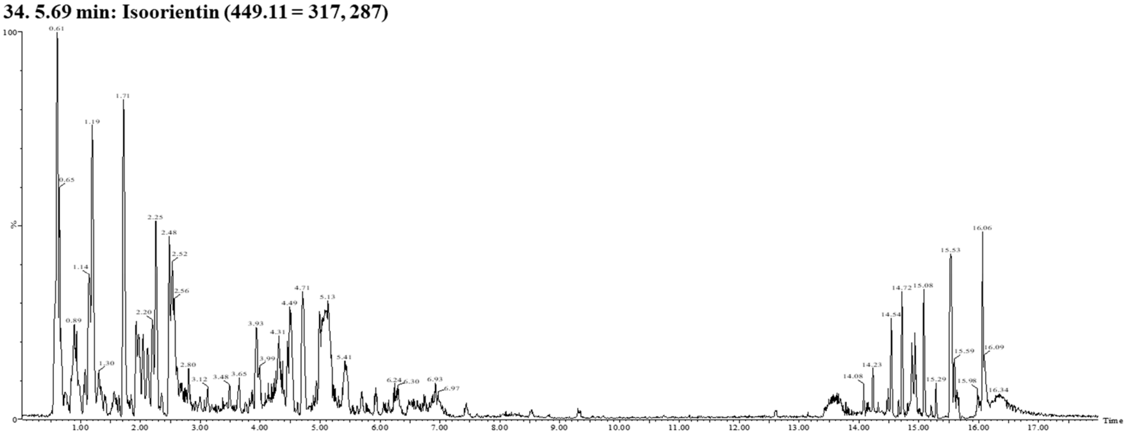

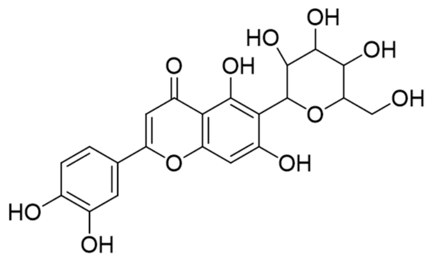

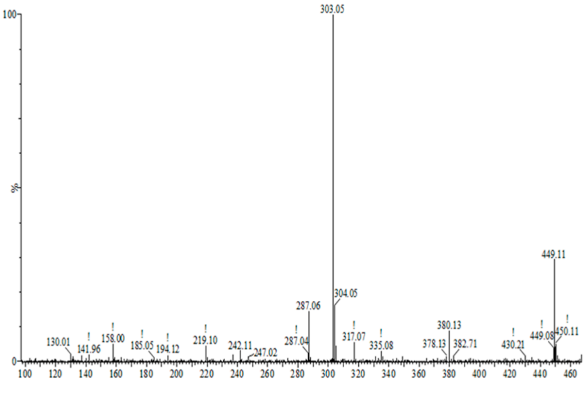

35. 14.23 min: Dehydrophytosphingosine (316.29 = 298, 280)

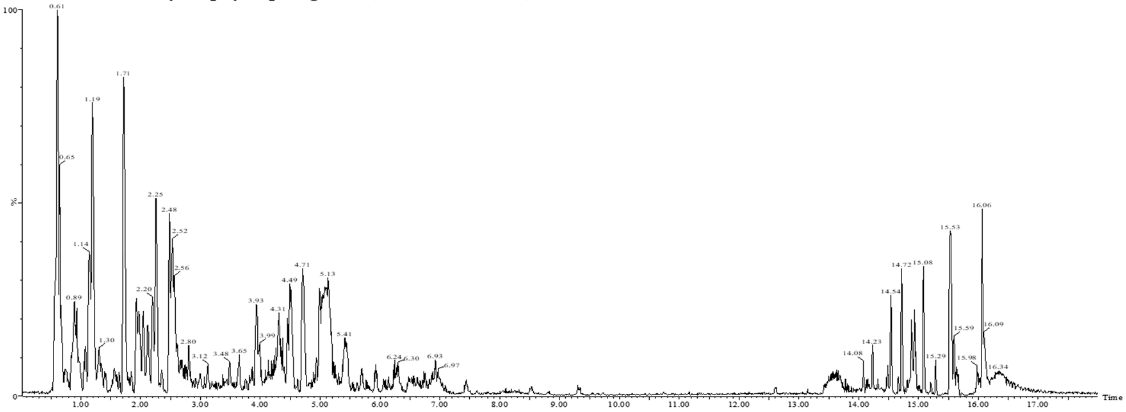

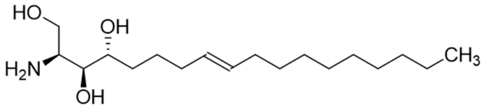

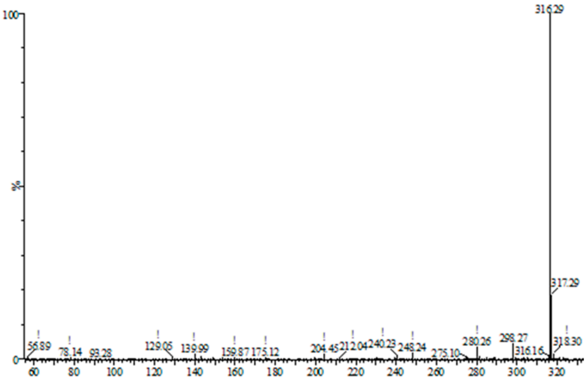

36. 14.48 min: LysoPC(18:3) (518.33 = 184)

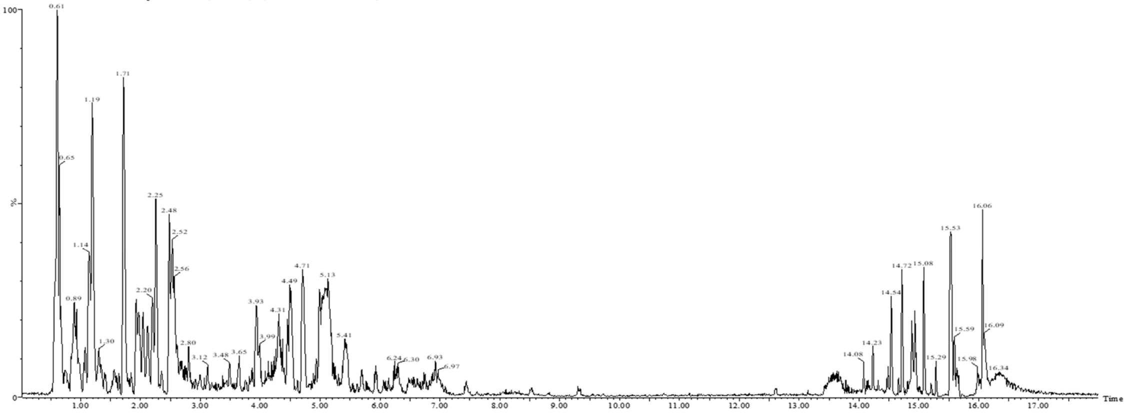

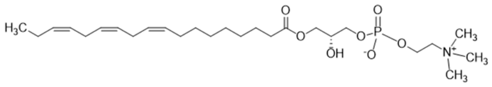

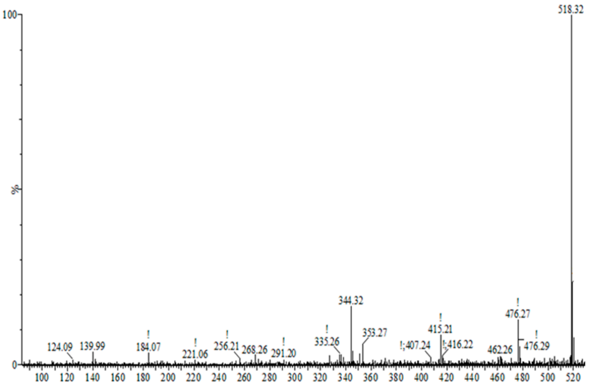

37. 14.65 min: LysoPC(18:2) (520.34 = 184)

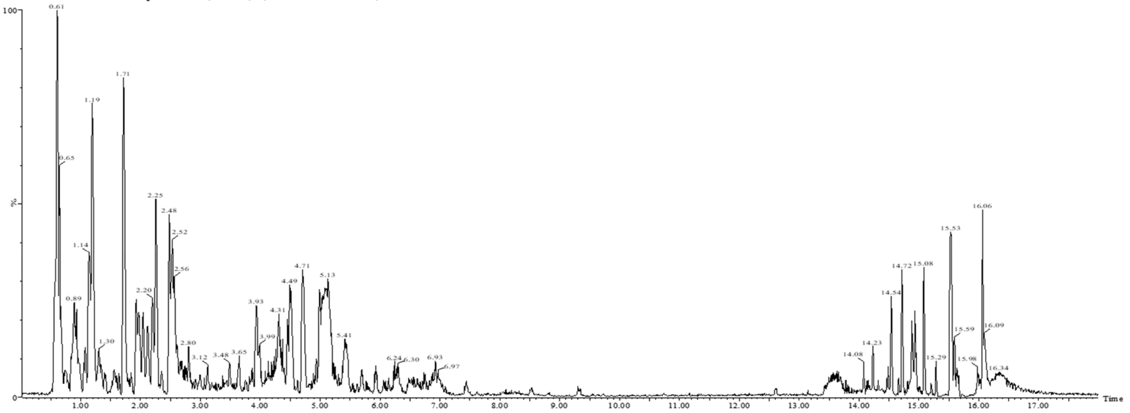

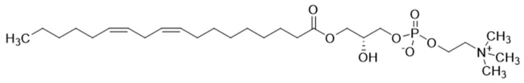

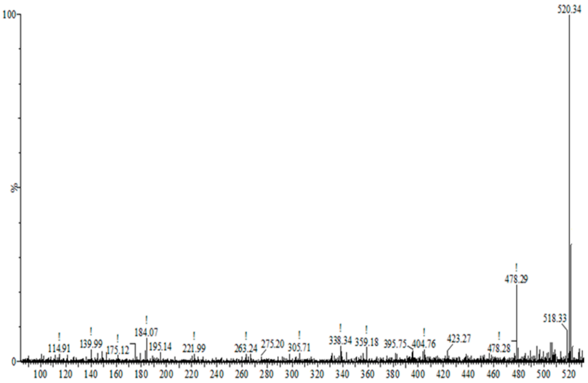

38. 14.89 min: LysoPC(16:0) (496.34 = 184)

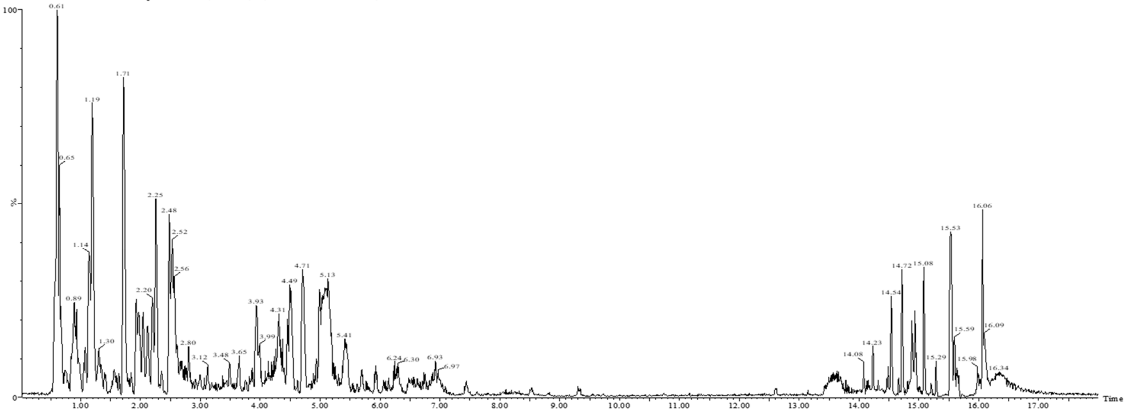

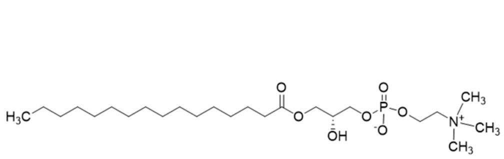

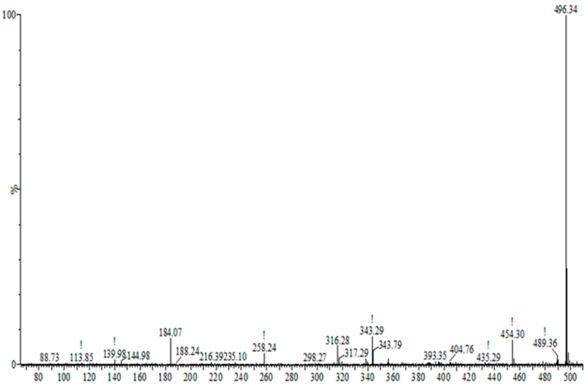

39. 14.94 min: LysoPC(18:1) (522.35 = 184)

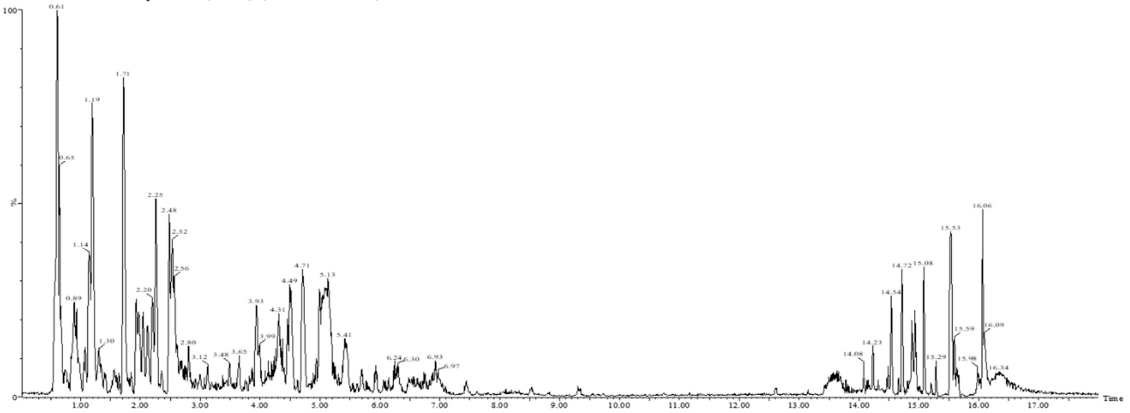

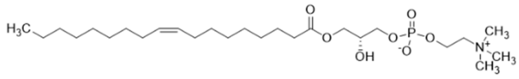

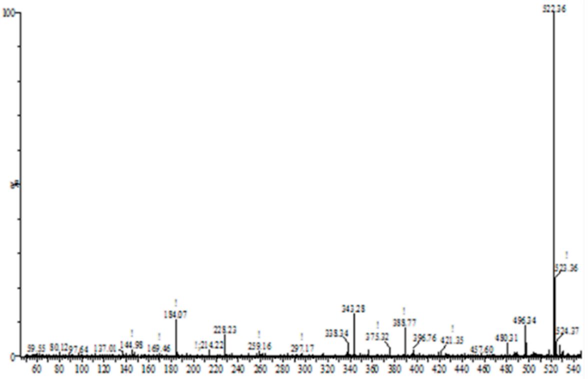

40. 15.08 min: Phytosphingosine-1-phosphate (398.23 = 280)

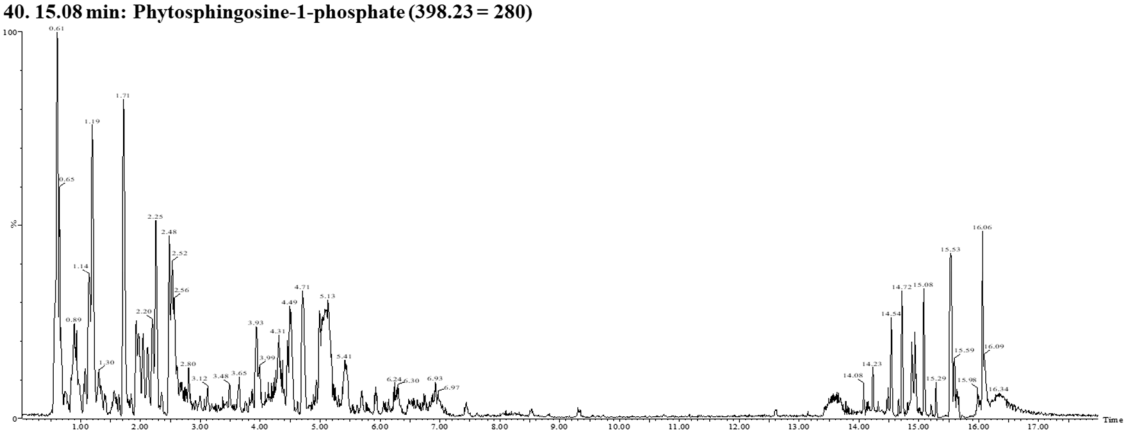

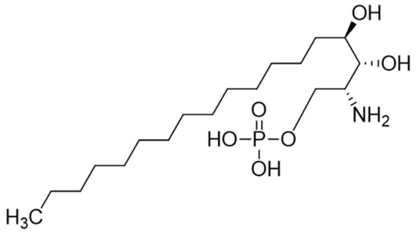

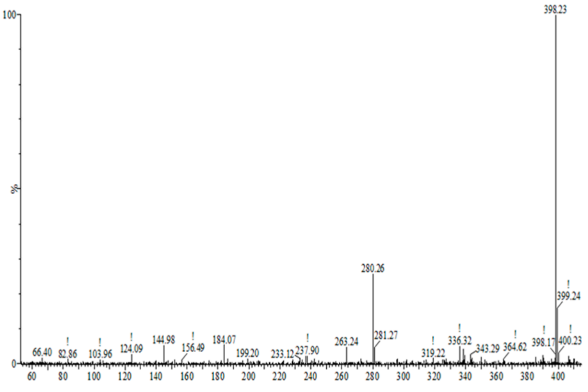

41. 15.20 min: Methyl phaeophorbide B (621.27 = 531, 487)

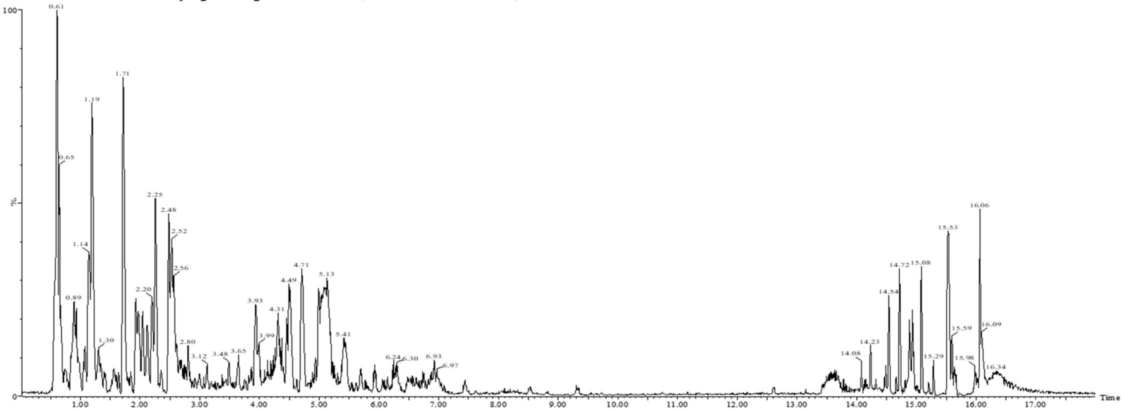

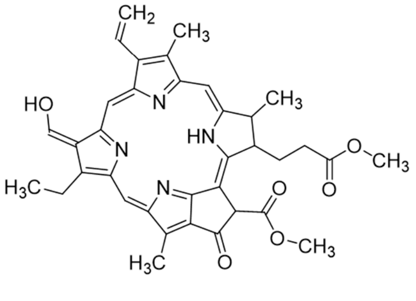

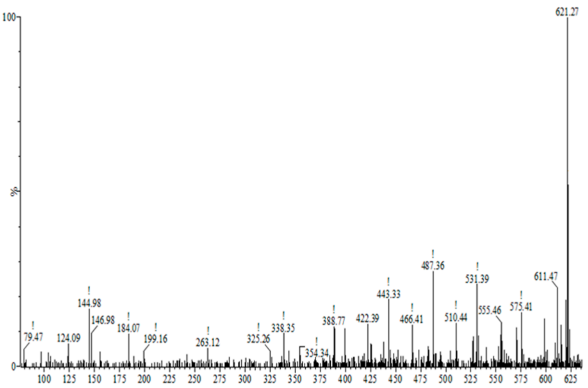

42. 15.29 min: Pheophorbide A (593.28 = 531, 487)

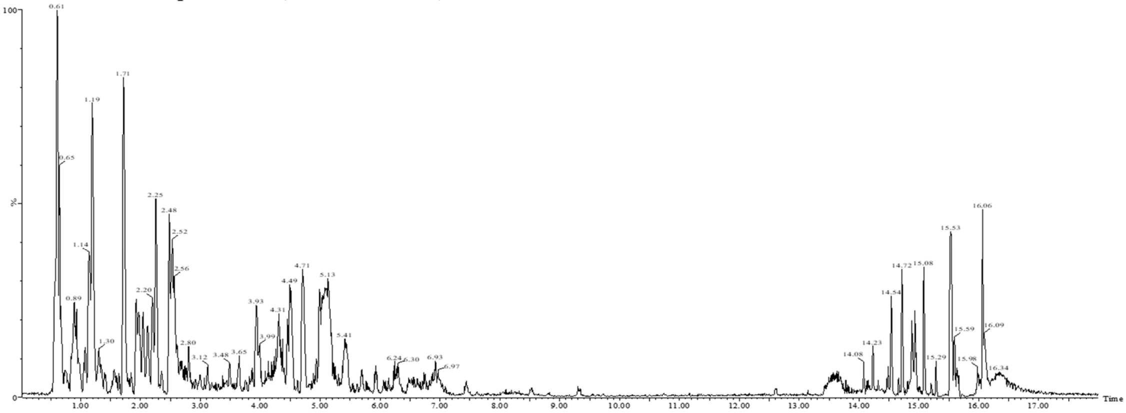

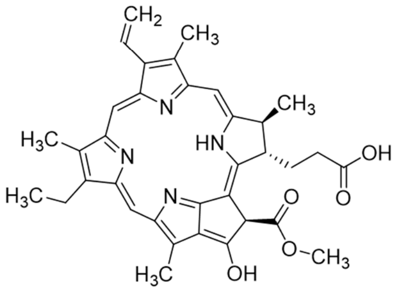

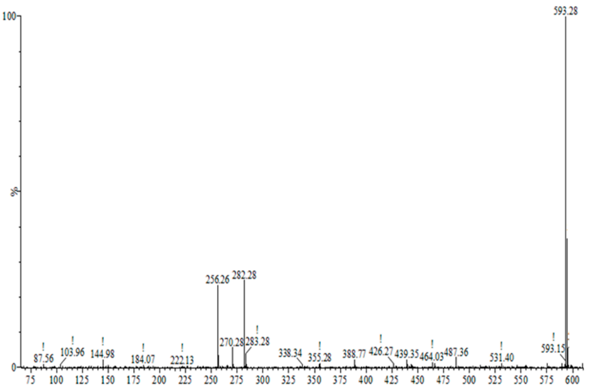

43. 15.53 min: Pheophorbide B (607.29 = 547)

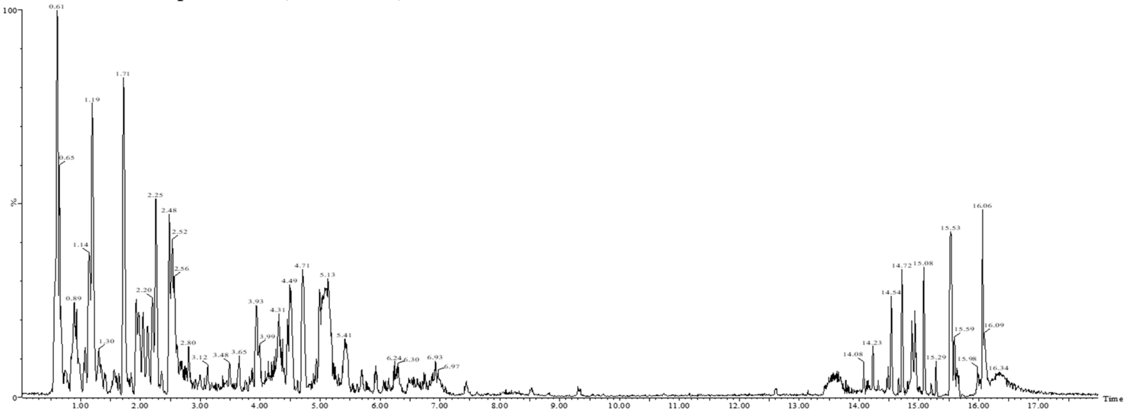

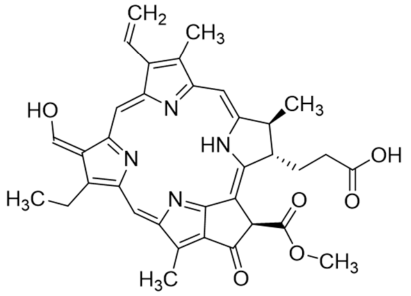

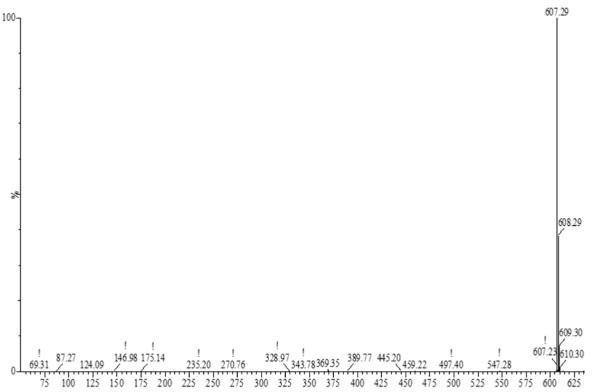

44. 16.06 min: PC(18:3/18:3) (778.54 = 184)

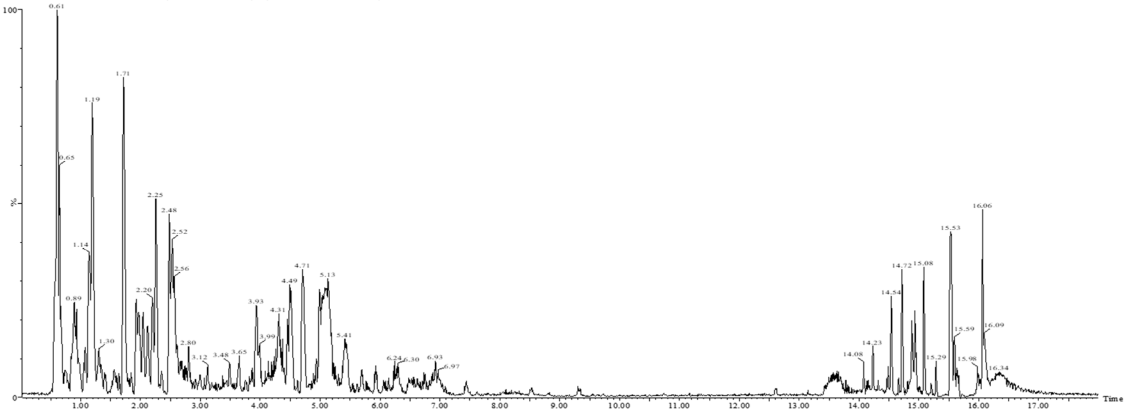

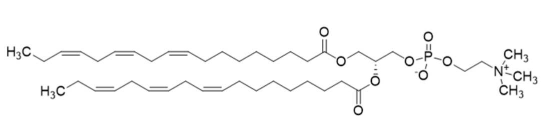

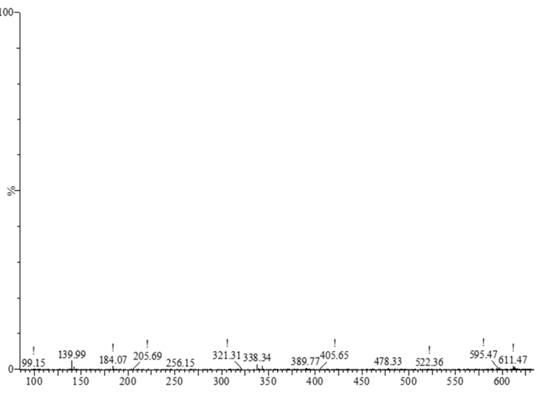

Figure S2. Photographs of the three plant-derived calli.

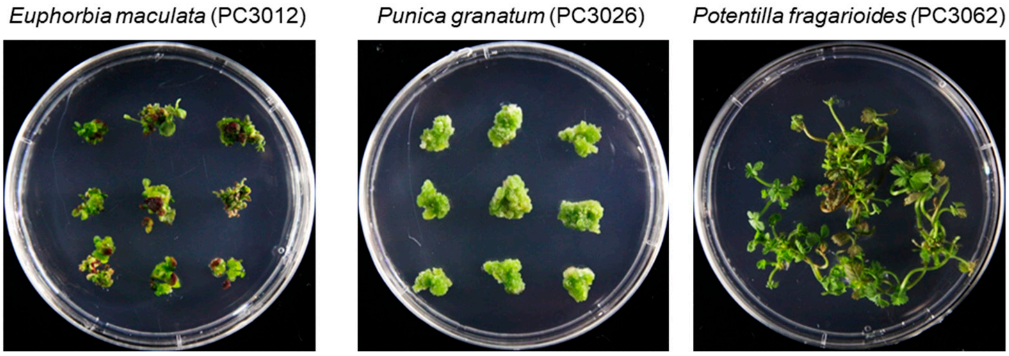

Table S1. Statistical analysis of *Euphorbia maculate* callus.

No.	Compounds	*P*-value	VIP
1	L -Arginine	4.8035E-05	1.1813
2	Pteroside B	2.5926E-03	1.1820
4	γ-Glutamyllysine	1.2853E-06	1.1869
5	Epicatechin 3-glucuronide	3.0743E-05	1.1827
6	*N*-(1-deoxy-1-fructosyl)phenylalanine	9.0207E-06	1.1851
7	L-Phenylalanine	3.0275E-04	1.1712
8	Sinapic acid	6.3085E-06	1.1857
9	Gallic acid 3-*O*-gallate	2.4184E-06	1.1866
11	*N*-(1-deoxy-1-fructosyl)tryptophan	3.2280E-04	1.1706
12	L-Trytophan	2.7843E-05	1.1829
13	2',7-Dihydorxy-4',5'-dimethoxyisoflavone	2.7938E-03	1.1364
14	Epicatechin 4'-glucuronide	4.8821E-04	1.1667
15	Quinic acid	1.1014E-03	1.1558
16	Chlorogenic acid	5.6233E-05	1.1807
17	Methyl gallate	4.3314E-03	1.1243
18	Caffeic acid ethyl ester	6.2065E-04	1.1643
19	Kaempferol 3-rhamnosyl-6''-(4''-acetylrhamnosyl)glucoside	2.1479E-04	1.1738
20	Acetylpterosin C	1.7324E-03	1.1476
21	1,2,3,4,6-Pentagallolyglucose	9.1111E-03	1.1673
22	Phenylalanylglycine	1.4533E-04	1.1763
24	Kaempferol 3-rhamnosyl-(6''-acetyl)galactosyl-7-glucoside	3.9069E-03	1.1275
25	Quercetin 3,7-diglucosyl-4''-galactoside	2.6206E-02	1.0274
26	Isoquercitrin	4.7637E-04	1.1668
29	Quercetin 3-(2''',6'''-digalloyl)galactoside	9.1116E-03	1.0946
30	Quercetin 3-(2-galloyl)glucoside	5.7370E-03	1.1738
31	Isorhamnetin 3-rutinoyl-4'-rhamnoside	1.9066E-02	1.0531
35	Dehydrophytophingosine	2.2181E-02	1.0405
36	LysoPC(18:3)	2.2775E-05	1.1834
37	LysoPC(18:2)	1.5407E-03	1.1838
38	LysoPC(16:0)	4.5405E-04	1.1863
39	LysoPC(18:1)	1.0885E-05	1.1849
42	Phaephoribide A	2.9379E-03	1.1353
44	PC(18:3/18:3)	1.4380E02	1.0723

VIP = variable importance in projection.

P-value = the significance of the difference between irradiated callus and non-irradiated callus
